# Early detection of cholera epidemics to support control in fragile states: estimation of delays and potential epidemic sizes

**DOI:** 10.1186/s12916-020-01865-7

**Published:** 2020-12-15

**Authors:** Ruwan Ratnayake, Flavio Finger, W. John Edmunds, Francesco Checchi

**Affiliations:** 1 Department of Infectious Disease Epidemiology, Faculty of Epidemiology and Population Health, London School of Hygiene and Tropical Medicine, London, UK; 2 Centre for the Mathematical Modelling of Infectious Diseases, London School of Hygiene and Tropical Medicine, London, UK; 3 Health in Humanitarian Crises Centre, London School of Hygiene and Tropical Medicine, London, UK; 4 Epicentre, Paris, France

**Keywords:** Armed conflict, Cholera, Communicable disease control, Epidemics, Outbreaks, Refugees, Surveillance

## Abstract

**Background:**

Cholera epidemics continue to challenge disease control, particularly in fragile and conflict-affected states. Rapid detection and response to small cholera clusters is key for efficient control before an epidemic propagates. To understand the capacity for early response in fragile states, we investigated delays in outbreak detection, investigation, response, and laboratory confirmation, and we estimated epidemic sizes. We assessed predictors of delays, and annual changes in response time.

**Methods:**

We compiled a list of cholera outbreaks in fragile and conflict-affected states from 2008 to 2019. We searched for peer-reviewed articles and epidemiological reports. We evaluated delays from the dates of symptom onset of the primary case, and the earliest dates of outbreak detection, investigation, response, and confirmation. Information on how the outbreak was alerted was summarized. A branching process model was used to estimate epidemic size at each delay. Regression models were used to investigate the association between predictors and delays to response.

**Results:**

Seventy-six outbreaks from 34 countries were included. Median delays spanned 1–2 weeks: from symptom onset of the primary case to presentation at the health facility (5 days, IQR 5–5), detection (5 days, IQR 5–6), investigation (7 days, IQR 5.8–13.3), response (10 days, IQR 7–18), and confirmation (11 days, IQR 7–16). In the model simulation, the median delay to response (10 days) with 3 seed cases led to a median epidemic size of 12 cases (upper range, 47) and 8% of outbreaks ≥ 20 cases (increasing to 32% with a 30-day delay to response). Increased outbreak size at detection (10 seed cases) and a 10-day median delay to response resulted in an epidemic size of 34 cases (upper range 67 cases) and < 1% of outbreaks < 20 cases. We estimated an annual global decrease in delay to response of 5.2% (95% CI 0.5–9.6, *p* = 0.03). Outbreaks signaled by immediate alerts were associated with a reduction in delay to response of 39.3% (95% CI 5.7–61.0, *p* = 0.03).

**Conclusions:**

From 2008 to 2019, median delays from symptom onset of the primary case to case presentation and to response were 5 days and 10 days, respectively. Our model simulations suggest that depending on the outbreak size (3 versus 10 seed cases), in 8 to 99% of scenarios, a 10-day delay to response would result in large clusters that would be difficult to contain. Improving the delay to response involves rethinking the integration at local levels of event-based detection, rapid diagnostic testing for cluster validation, and integrated alert, investigation, and response.

## Background

Cholera transmission was reported in 34 countries in 2018 and 55 countries in 2019 [1, 2]. The disease is estimated to be substantially under-recorded [3]. Large cholera epidemics frequently coincide with armed conflict and humanitarian crises, including those in Democratic Republic of the Congo, Iraq, Somalia, South Sudan, and Yemen [4–8]. At the start of a cholera outbreak, transmission is driven by the low capacity to detect and isolate the first identified cases. Inadequate preparedness and poor access to case management drives increased mortality. The rapid detection and control of small outbreaks is therefore key for efficient control before an epidemic propagates [9].

In 2017, the Global Task Force on Cholera Control (GTFCC) recommended that countries increase their capacity to contain small outbreaks, using rapid response teams, to aid efforts to substantially reduce global transmission by 2030 [10]. However, little is known about the global capacity for rapid detection of, and response to, cholera outbreaks. In a review of the detection of all-pathogen outbreaks in Africa reported in the World Health Organization’s (WHO) Disease Outbreak News from 1996 to 2014, the median time from onset of symptoms of the first identified case (or health facility visit, if unavailable) to discovery of the outbreak (defined, for example, as the declaration of the outbreak or appearance in an official report) was 27 days [95% CI 20–31.5] [11]. A review of all-pathogen outbreaks in fragile and conflict-affected states from 2000 to 2010 found a similar median delay of 29 days [range 7–80] from symptom onset of the first identified case to detection of the outbreak and a median delay of 7 days [range, 0–30] from detection to investigation [12]. For cholera, a month-long delay in detection represents approximately 6 median serial intervals and, thus, a high potential for uncontrolled transmission [13].

To understand the potential for early detection and rapid response for cholera outbreaks in fragile and conflict-affected states, we examined temporal trends in cholera epidemics to evaluate with what delays the first case or cluster presented, was detected, investigated, responded to, and was confirmed by laboratory culture. We modeled epidemic sizes corresponding to these delays. To explain these delays, we investigated the mechanisms for early warning of these outbreaks, predictors of delays, and global improvements in reducing delays.

## Methods

### Compilation of cholera outbreaks

The period of 2008 to 2019 was chosen to reflect recent experience with cholera response. A list of countries that appeared ≥ 2 times during this period on the World Bank’s Harmonized List of Fragile Situations, and had a documented cholera burden as per the GTFCC’s 2017 list of cholera-affected countries, was compiled (Additional file 1) [10, 14]. Small-island states affected mainly by climate change rather than conflict were excluded. A list of countries meeting the fragility criteria but not included in the GTFCC list were included if they were documented using other sources as having had cholera outbreaks from 2008 to 2019 (i.e., Iraq, Myanmar, and Syria). Cholera-affected countries that did not meet the fragility criteria but either (a) hosted refugees (i.e., Kenya, Tanzania, and Uganda) and/or (b) border fragile and conflict-affected states with cholera outbreaks (i.e., Benin, Ethiopia, Niger, Nigeria, Tanzania, and Zambia) were included. Given that no annual list of annual cholera outbreaks exists, a list of outbreaks was compiled using a two-step process. We first reviewed the WHO’s annual cholera reports to identify which countries reported transmission during the study period [15]. Countries that do not routinely report cholera to WHO but are known from other sources to have had cholera outbreaks were included (e.g., Ethiopia and Myanmar). We then sought details on the occurrence of sub-national outbreaks from the WHO’s Disease Outbreak News and UNICEF’s Cholera Outbreaks in Central and West Africa Bulletin (2015–9) [16, 17]. The GTFCC definition of a cholera outbreak was applied (cholera-free region: ≥ 1 culture-confirmed case and evidence of local transmission, or year-round transmission: unexpected increase in magnitude or timing of suspect cases over 2 weeks with laboratory confirmation) [18]. As stool sampling and transport is often unfeasible in insecure settings, we included instances where cholera alerts were identified (e.g., one case of acute watery diarrhea (AWD) testing positive for cholera by rapid diagnostic test (RDT)) [19]. Finally, we included cholera alerts that triggered the cholera investigation mechanism, but where testing detected another pathogen, in order to explore detection and investigation mechanisms.

### Compilation of reports on cholera outbreaks

We searched the peer-reviewed literature for further identification and reporting on cholera outbreaks. Peer-reviewed articles were sourced from PubMed/MEDLINE using a date-specific keyword search (“country AND cholera”). Given that only a small number of outbreaks are reported in the scientific literature, we searched the gray literature, including epidemiological summaries, national cholera preparedness and response plans, and non-peer-reviewed studies. The sources included the following: (1) Reliefweb (https://reliefweb.int/, a repository of documents and data from humanitarian crises) using a date-specific keyword search (“country AND cholera”; “UNHCR AND cholera”) and (2) regional outbreak bulletins and journals including the WHO EMRO Weekly Epidemiological Monitor (2008 to 2019), WHO AFRO Outbreaks and Emergencies Bulletin (2017–9), WHO SEARO Journal of Public Health, WHO WPRO Western Pacific Surveillance and Response Journal, and UNICEF Cholera Outbreaks in Central and West Africa Bulletin (2015 to 2019) [16, 20–23]. The Program for Monitoring Emerging Diseases (ProMED) database of disease observations from media sources was used to fill in missing information on dates, but was not used as the primary source of information [24]. When little information was available from the sources above, websites of ministries of health and crisis-specific surveillance systems (e.g., early warning alert and response systems or networks (EWARS/EWARN) or disease early warning systems (DEWS)) were searched. An example includes the EWARN of the Syrian Assistance Coordination Unit for Northern Syria [25].

### Inclusion criteria and data extraction

Outbreaks were included if at least two of the following dates were available: (1) dates of symptom onset of the primary case, and/or case presentation, and/or outbreak detection, and (2) dates of investigation and/or response. If the date of symptom onset for the primary case was missing, it was estimated as 5 days before the date of case presentation (equal to the median delay for outbreaks with available date of symptom onset), or date of outbreak detection if date of case presentation was unavailable. If the date of case presentation was unavailable, it was replaced by the date of outbreak detection. The earliest dates of (1) symptom onset for the first identified primary case, (2) case presentation to a health facility, (3) detection of outbreak/alert raised, (4) investigation by local health authorities, (5) response, and (6) laboratory confirmation by culture were extracted (Table 1). We defined the date of response as the earliest date by which a cholera-specific control measure was applied to the outbreak-affected area (e.g., water, sanitation, and hygiene (WASH) activities, setup of case management, active case-finding, community-based activities, and delivery of cholera kits). The starting month and year of the outbreak, geographical context (i.e., urban, rural, or displacement camp), type of crisis or fragility (i.e., armed conflict, fragile state, natural disaster, refugee setting, non-fragile state bordering a fragile state), and the WHO region were extracted. Any additional information on factors that may have contributed to the observed delay, including presence of an early warning function, was extracted. Details on the signal type for outbreak detection were recorded, if available, as a (1) *formal alert* detected by health workers reported immediately within the surveillance system, (2) *informal alert* from community members or a non-governmental organization (NGO) reported immediately, or (3) *weekly data analysis* of surveillance trends.

**Table 1:** Dates used to estimate delays in detection, investigation, and response for cholera outbreaks

Date	Defined as earliest date (by priority)
Date of start of outbreak	1. Symptom onset for first identified case 2. Case presentation to health facility (less 1 day)
Date of alert/outbreak detection	Alert issued from health facility, community health worker, community member, local public health office, or laboratory
Date of investigation	Investigation by local authorities
Date of earliest response	Any cholera-specific response activity (case-finding, control measures by health facility or public health office, household/community WASH, case management)
Date of laboratory confirmation	First documented culture confirmation

### Analysis of delays and their predictors

For each outbreak, median delays and their interquartile ranges (IQR) were calculated by subtracting the date of symptom onset of the primary case from the dates of (1) case presentation, (2) outbreak detection, (3) investigation, (4) earliest response, and (5) laboratory confirmation. For each outbreak, the dates were graphed on a timeline.

To investigate the association between the observed delay from symptom onset of the primary case to response and potential predictor variables, a multivariate ordinary least-squares regression model was used. Delay to response was log-transformed to produce a normalized distribution. Extreme values in delay to response were judged to represent meaningful delays rather than data errors and were retained in the dataset. Predictor variables included signal type, context, crisis, WHO region, and year of outbreak onset (to detect any secular trend). Akaike information criterion (AIC) and a step-wise selection process was used to assess model fit and complexity. In separate regressions, year of outbreak onset was used as a predictor variable to investigate secular trends for delays to case presentation, outbreak detection, investigation, and confirmation. Loess curves were used to visualize the temporal trends using a smoothed trend line that down-weighted extreme values [26]. Percent change and 95% confidence intervals were presented for each regression.

### Branching process model

A preliminary review of retrieved reports demonstrated that the early epidemic sizes at the dates that the outbreak was detected and responded to were rarely documented. Instead, to estimate the potential early epidemic sizes at each delay, a branching process model was used to estimate the median and range of epidemic sizes at the time points indicated by the median delays to case presentation, investigation, response, and confirmation [27–29]. We simulated multiple outbreaks using 10,000 runs and calculated the proportion of these outbreaks with early epidemic sizes below the threshold of 20 cases in a 5 to 30 day period. We selected this threshold arbitrarily as the outbreak size with potential to be contained. Transmission started with a seed case(s) which generated secondary cases from a negative binomial distribution *Z* ~ NegB(*R*_E_, *k*) with a mean equivalent to the reproduction number (*R*_E_, 2.5 [13, 30], reflecting early and high transmission potential among an unvaccinated population) and heterogeneity introduced by a dispersion parameter (*k*, 4.5, reflecting low overdispersion in *R*_E_) [31]. Each new infection drew a time of infection from a serial interval distribution *S* ~ gamma(shape = 0.5, rate = 0.1) with a median of 5 days [4, 13, 32]. We assumed that at the time of outbreak detection there were 3 seed cases and that all resulting infectious persons were symptomatic. Simulations would end by chance when either the cases did not produce additional secondary cases, or they reached 1000 cases (representing a large outbreak). In a sensitivity analysis, we considered outbreaks of larger size at detection (i.e., 10 and 20 seed cases).

All analyses were carried out in R statistical software version 4.0.3 [33].

## Results

### Description of outbreaks

Seventy-six outbreaks from 34 countries met the inclusion criteria. Overall, 1970 documents were reviewed, and 138 documents were retained (1–4 documents per outbreak including 28 peer-reviewed articles and 110 gray literature sources as listed in Additional file 2) [34–171]. Where countries reported acute watery or severe diarrhea as a proxy for cholera (e.g., Ethiopia, Myanmar) [172, 173] or where surveillance was poor due to conflict in remote areas (Myanmar, Northern Nigeria, and Syria as documented by Sparrow and colleagues) [19], few or no reports were located. Few reports from endemic countries with ongoing transmission (e.g., Cameroon, Democratic Republic of the Congo, Somalia) were found, likely due to the difficulty in ascertaining start dates. Three false alerts resulting in the exclusion of cholera were identified in Cameroon (2) and Syria (1); we described these outbreaks qualitatively and left them out of the quantitative analysis [100, 101, 108]. One alert of an RDT-positive case where culture could not be obtained due to ongoing conflict was identified in Syria and kept in the quantitative analysis (noting that confirmation was not possible) [83, 84]. Fifty-one (67%) of the 76 outbreaks were missing the date of onset of symptoms for the primary case.

Narrative descriptions and sources of information for outbreaks are compiled in Additional file 2. Most reports were from Africa (80.3%, mainly Chad, South Sudan, Burundi, and Uganda) and the Eastern Mediterranean region (13.2%, mainly Yemen, Iraq, and Syria) (Table 2). Outbreaks occurred during armed conflicts (e.g., Afghanistan, South Sudan, Yemen), after natural disasters (e.g., cyclones in Mozambique, post-earthquake in Nepal), in fragile situations (e.g., Angola, Chad), in refugee settlements (e.g., camps in Kenya and Tanzania), and in countries bordering cholera-affected fragile states (e.g., Benin, Tanzania). Most reports (56.6%) were from urban sites. Where the information was available (55/76 outbreaks), most (83.6%) were detected through formal and informal alerts compared with weekly data analysis (16.4%).

**Table 2:** Characteristics of outbreaks, 2008–2019

Characteristic	*N* (%)
**WHO region**	
Africa (AFRO)	61 (80.3)
Eastern Mediterranean (EMRO)	10 (13.2)
South-East Asia (SEARO)	3 (4.0)
Americas (PAHO)	1 (1.3)
Western Pacific (WPRO)	1 (1.3)
**Context**	
Urban	43 (56.6)
Rural	27 (35.5)
Refugee or displacement camp	6 (7.9)
**Crisis**	
Fragile situation	31 (40.8)
Armed conflict	25 (32.9)
Country bordering FCAS	10 (13.2)
Natural disaster	5 (6.6)
Refugee setting	5 (6.6)
**Surveillance system** *N = 47 (not reported for 29 outbreaks)*	
Early warning function	36/47 (76.6)
Through routine surveillance	22/36 (61.1)
EWARS/DEWS	14/36 (38.9)
Routine surveillance	11/47 (23.4)
**Signal** *N = 55 (not reported for 21 outbreaks)*	
Alert	46/55 (83.6)
Formal alert	37/46 (80.4)
Informal alert	9/46 (19.6)
Weekly data analysis	9/55 (16.4)

### Delays and potential epidemic sizes

**Fig. 1:**
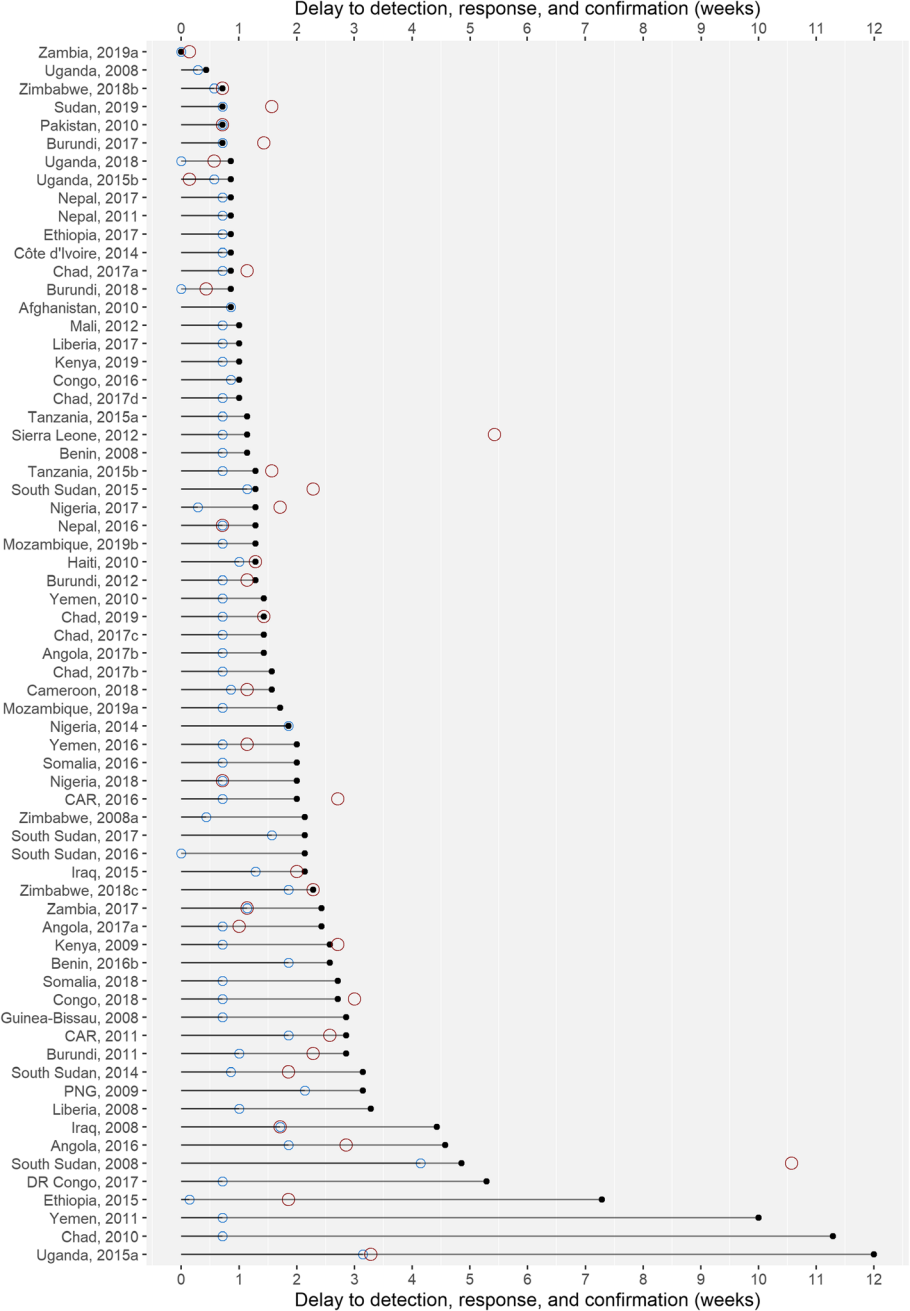
Delay in weeks from date of onset of symptoms to outbreak detection (blue circle), response (black circle), and confirmation (red circle), by outbreak, 2008–2019 (excluding outbreaks missing response date)

Median delays are listed in Table 3, and histograms of the delays are listed in Additional file 3. The timelines of the individual outbreaks are visualized in Fig. 1. The median delay from date of the first identified case’s symptom onset to case presentation at the health facility was 5 days (IQR 5–5). The median delays between symptom onset of the primary case and detection (5 days, IQR 5–6), investigation (7 days, IQR 5.8–13.3), and response (10 days, IQR 7–18) spanned 1 to 2 weeks. Across countries, these delays varied; investigations and responses were routinely launched on the same or next day in Cameroon and Nepal, while long delays of 70 days in Uganda in 2015, 79 days in Chad in 2010, and 84 days in Yemen in 2011 were reported. The median delay to laboratory confirmation, for the 41/76 outbreaks for which the information was available, was 11 days (IQR 7–16), similar to the delay to response. Countries affected by conflict frequently had delays from symptom onset of the primary case to response greater or equal to 2 weeks (Fig. 1).

**Table 3:** Median delays (with interquartile range (IQR) and range)

Delay	Median delay (days) (IQR)	Range (days)
Delay to case presentation (*n* = 76)		
Symptom onset to case presentation	5 (5–5)	0–22
Delay to detection (*n* = 76)		
Symptom onset to outbreak detection	5 (5–6)	0–29
Case presentation to outbreak detection	0 (0–0.3)	0–24
Delay to investigation (*n* = 48)		
Symptom onset to investigation	7 (5.8–13.3)	0–84
Case presentation to investigation	2 (1–8)	0–62
Delay to response (*n* = 67)		
Symptom onset to response	10 (7–18)	0–84
Case presentation to response	6 (2.5–13.5)	0–74
Delay to confirmation (*n* = 41)		
Symptom onset to confirmation	11 (7–16)	0–74

Several outbreaks were detected when already-large, challenging containment (e.g., Afghanistan, 2011, 255 cases, ID 2; Chad, 2017, 50 cases and 13 deaths, ID 19; Ethiopia, 2015, 268 cases, ID 28; Haiti, 2010, > 1000 cases, ID 32). Table 4 summarizes the model-simulated early epidemic sizes that the outbreaks could have reached by the date of different delays and different initial outbreak sizes. With 3 seed cases at detection, a median delay to case presentation of 5 days resulted in a median epidemic size of 9 cases (upper range, 29 cases), with nearly all outbreaks < 20 cases (98.6%). A median delay to response of 10 days resulted in a median epidemic size of 12 cases (upper range, 47 cases), with a comparable proportion of outbreaks < 20 cases (92.6%). Lengthening the delay to response to 30 days resulted in an upper range of 72 cases, with 67.7% of outbreaks remaining < 20 cases. Using 10 seed cases to simulate outbreaks of larger size at detection, a median delay to case presentation of 5 days resulted in a median epidemic size of 28 cases (upper range, 55 cases), with a minority of outbreaks < 20 cases (5.7%). With a median delay to response of 10 days delay, there was a median epidemic size of 34 cases (upper range, 67 cases), with < 1% of outbreaks < 20 cases. At 30 days, the upper range was 100 cases, with < 1% of outbreaks remaining < 20 cases. With 20 seed cases at detection, outbreaks enlarged quickly, reaching a median of 55 cases (range 30–89) with a median delay to response of 5 days and median of 65 cases (range 40–110) with a median delay to response of 10 days.

**Table 4:** Simulated epidemic sizes (with standard deviation (SD) and range), and proportion of outbreaks < 20 cases for outbreaks of 3, 10, and 20 seed cases at detection

		3 seed cases		10 seed cases		20 seed cases
Delay from onset of symptoms (primary case)	**Median delay (days)**	**Median epidemic size (SD, range)**	**% outbreaks < 20 cases**	**Median epidemic size (SD, range)**	**% outbreaks < 20 cases**	**Median epidemic size (SD, range)**
Case presentation or outbreak detection	5	9 (3.7, 3–29)	98.6	28 (5.7, 12–55)	5.7	55 (7.4, 30–89)
Investigation	7	10 (4.4, 3–40)	96.9	31 (6.1, 11–61)	1.6	60 (8.2, 34–99)
Response	10	12 (5.1, 3–47)	92.6	34 (7.0, 16–67)	< 1	65 (8.8, 40–110)
Confirmation	11	12 (5.4, 3–50)	91.9	35 (7.1, 13–69)		67 (9.1, 41–105)
Delay from onset of symptoms (primary case)	**Counterfactual delay (days)**	**Median epidemic size (SD, range)**	**% outbreaks < 20 cases**	**Median epidemic size (SD, range)**	**% outbreaks < 20 cases**	**Median epidemic size (SD, range)**
14-day delay	14	14 (6.0, 3–51)	85.8	37 (7.8, 17–79)	< 1	70 (9.7, 41–113)
21-day delay	21	16 (7.4, 3–63)	76.6	40 (9.0, 16–87)		74 (10.8, 43–124)
30-day delay	30	18 (8.9, 3–72)	67.7	43 (10.3, 18–100)		78 (12.2, 43–131)

Legend: *DRC* Democratic Republic of the Congo, *CAR* Central African Republic, *PNG* Papua New Guinea

### Factors associated with delays

**Fig. 2:**
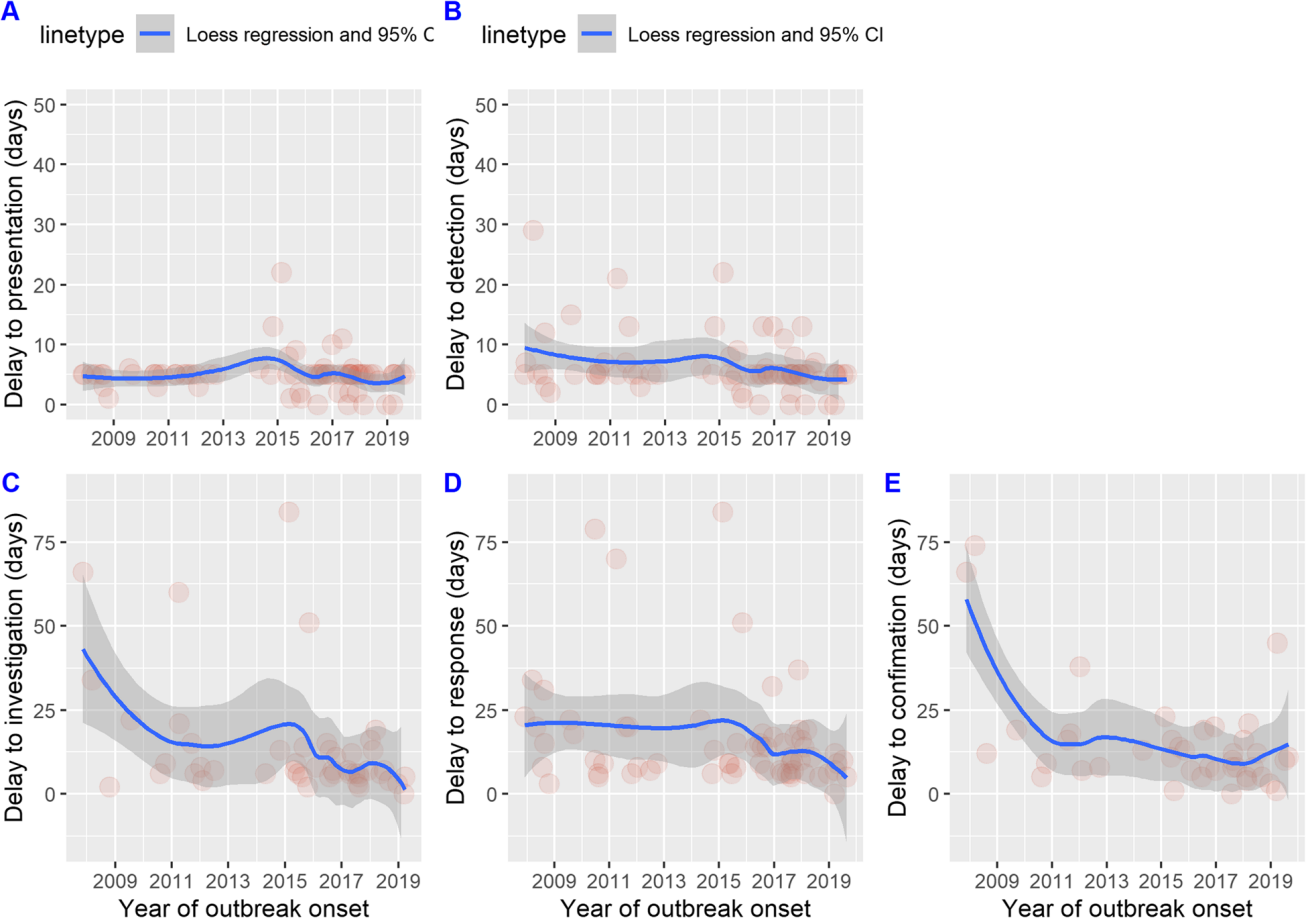
Scatterplot of cholera outbreaks by delay between date of symptom onset of the primary case and dates of **a** presentation, **b** detection, **c** investigation, **d** response, and **e** confirmation, and Loess curves, as a function of outbreak start date, 2008 to 2019. Red dots represent individual outbreaks over time and gray shading indicates 95% CI of Loess regression

**Fig. 3:**
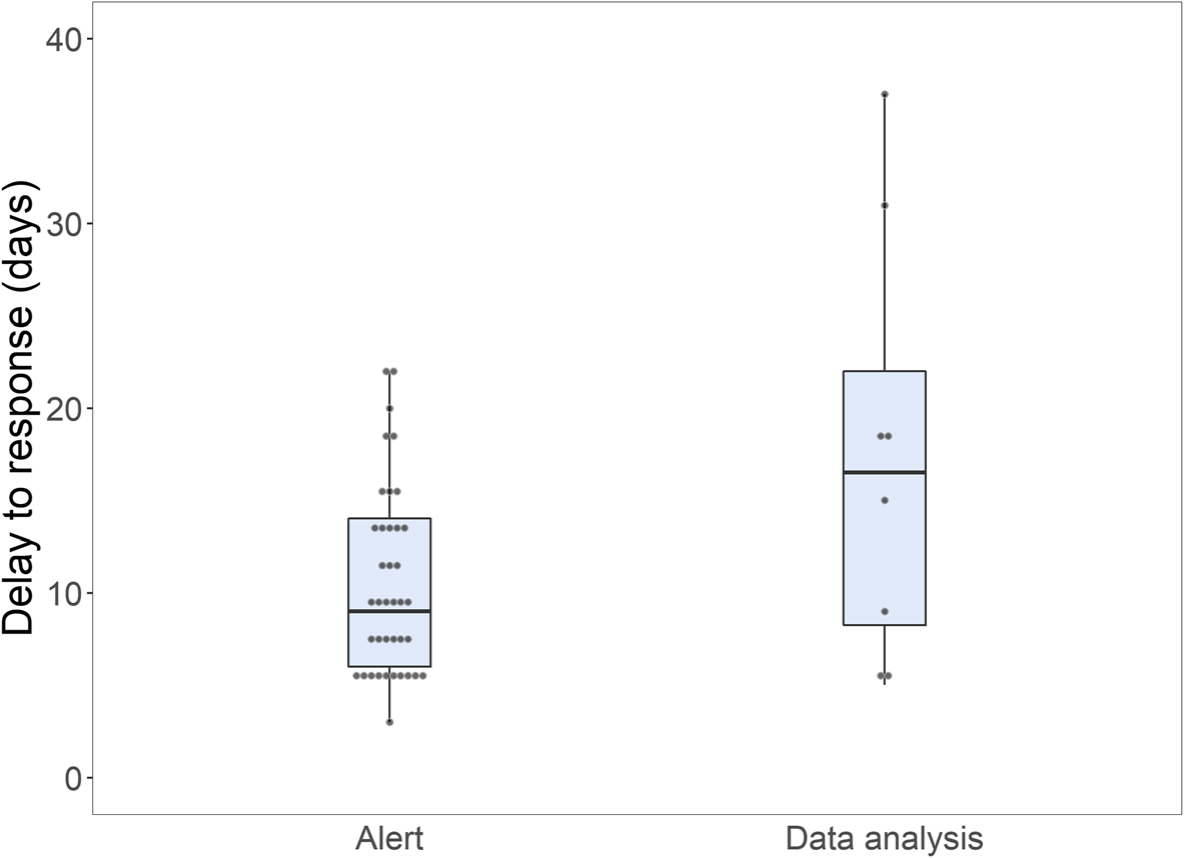
Delay from onset of symptoms of the primary case to response, by signal type (immediately-notified alert compared with weekly data analysis), 2008–2019 (*N* = 49/76 outbreaks with information on signal type available). Gray dots represent individual outbreaks

Given that the signal type was complete for only 55/76 observations, two models were implemented: a multivariable adjusted model (including year of outbreak onset, WHO region, context, and crisis type), and a bivariate model for signal type only (informal/formal alert versus weekly data analysis). Using AIC for the multivariable model, including only year of outbreak onset returned the lowest AIC score (Additional file 4). A weak crude association between year of outbreak onset and delay to response, with an annual decrease in response time of 5.2% (95% CI 0.5–9.6, *p* = 0.03), was found (visualized in Fig. 2). This model met the assumptions for linearity and homogeneous variance and explained 6% of the variance. Similar decreases in delay to detection, investigation, and confirmation were found (Fig. 2 and Additional file 5). In the second model, alerts (versus data analysis) were associated with a reduction in response time of 39.3% (95% CI 5.7–61.0, *p* = 0.03) (boxplot displayed in Fig. 3). The model met the assumptions for ordinary least-squares regression but one extreme value in delay affected the leverage. The model explained 8% of the variance.

More information from the examination of outbreaks is illustrative of the use of alerts. Of the 83.6% of outbreaks detected through alerts, 37/46 (80.4%) were through alerts by a health worker or community health worker and 9/46 (19.6%) through informal alerts by community members. For example, in 2015, in Aleppo, Syria, an alert was issued through the EWARN via phone after RDT testing of a suspect, and an investigation initiated based on the positive result (2-day delay to investigation, ID 63). In 2017, in a displacement camp in Northern Nigeria, an alert of a suspect case was issued by MSF by phone through the EWARS on the same day of case presentation (2-day delay to investigation, ID 48), demonstrating the rapid recognition of a suspect case by health workers [171]. Comparisons of outbreaks within countries are instructive. In Benin, of two outbreaks in rural areas detected in 2016, one outbreak was detected through an immediate call to public health authorities (5-day delay to detection, ID 7) while for the other outbreak, issuance of the alert on the weekly set day of routine surveillance data transmission resulted in a 13-day delay to detection (ID 8). In Central African Republic in 2011, an alert from the community of multiple suspect cases was issued late (13-day delay, ID 16) compared with 2016, when an alert from Red Cross volunteers was issued in half the time (5-day delay, ID 17).

In several instances, early warning systems further benefited from rapid investigation and response. For example, in Afghanistan, in 2010, the DEWS provided a response mechanism to link the detection of a large cluster of 60 suspect cases in a remote and insecure village by a local NGO with rapid action which reportedly led to containment within a month (6-day delay to investigation, ID 1). In 2011, the DEWS in Afghanistan detected an already-large outbreak of 255 suspect cases in multiple clusters but with a rapidly administered response (21-day delay to investigation, ID 2). Reduced transmission within 3 months followed. In Liberia in 2017, a suspect case that died en route to the health facility was detected based on symptoms, triggering a rapid response to isolate additional cases in the index case’s village (7-day delay to investigation, ID 38). In Chad in 2017, two suspect cases among children which resulted in rapid progression to death were reported to the local health facility, who investigated the source village and found a larger cluster of 50 cases and 13 deaths (6-day delay to investigation, ID 19). Though already a large outbreak, this led to a response on the following day.

Information in reports suggested improvements in surveillance, investigation, and response over time. In Cameroon in 2016, two false alerts for cholera later attributed to food poisoning and rotavirus were made by health workers and community members respectively and led to rapid investigation upon detection, testing by RDT and culture, and ongoing control activities during the investigation period (ID 13, 14). In Somalia, faster response in insecure urban areas using EWARS in 2016 and 2018 can be compared to a lack of a comprehensive early response during ongoing transmission over 2 months in 2008 (14- and 19-day delays versus 2-month delay, ID 54–56). Nepal’s EWARS facilitated rapid detection and response to clusters from 2011 onwards (total delays 6–9 days, ID 42–44) [75, 130, 162]. In 2016, RDT capacity was added at health facilities to enable better discrimination between alerts of cholera or diarrhea due to other pathogens [161].

### Potential factors related to long delays to response

Long delays from symptom onset of the primary case to response (~ 2 weeks) were observed in 29/67 (43.2%) outbreaks for which a response date was available. These appeared to be related to poor sensitivity of the formal surveillance system due to the remote locations of outbreaks [63] (Papua New Guinea, ID 52); insecurity posed by armed conflict (Somalia, 2008, ID 54; South Sudan, 2008, ID 57; Yemen, 2011, ID 72); reliance on laboratory confirmation to declare an outbreak before initiating a comprehensive response (Iraq, 2008, 2015, ID 33, 34, South Sudan, 2014, ID 58); assuring government declaration and mobilization of non-governmental actors (CAR, 2011, ID 16); a less effective local response which required reinforcement by capacity from the national level or other partners (Congo, 2018, ID 25; Ethiopia, 2015, ID 28; Guinea-Bissau, 2008, ID 31; South Sudan, 2017, ID 61; Uganda, 2015, ID 68; Zimbabwe, 2018, ID 79) [113]; and missed superspreading events (e.g., a funeral in Zimbabwe, 2018, ID 79) [136].

## Discussion

In an era of large-scale cholera epidemics in conflict settings like Yemen and previously cholera-free settings like Haiti, improving and sustaining early detection and response to small outbreaks remains critical for averting large-scale epidemics. Reducing delays in the timelines of patients presenting to health facilities, increasing capacity of health workers to recognize suspect cases of cholera, and reinforcing local investigation and response therefore remain as important as vaccination and other emerging tools. Some of the largest outbreaks in recent years in South Sudan (2014–6), Ethiopia (2015 onwards), and Zambia (2017–8) have suffered from late detection and/or response, which has led to surges of cases that have overwhelmed health systems [131, 140, 174–176].

Our findings indicate that from 2008 to 2019, median delays from symptom onset of the primary case to case presentation at the health facility and to response were approximately 5 days and 10 days, respectively. Longer delays to response were documented across the whole time period. Evaluations from Nigeria, Yemen, and other settings have shown that reasons for delays to detection include poor population access to health services due to disrupted health systems and/or insecurity, difficulty in discerning diarrhea and dehydration due to cholera from other causes without rapid diagnostics, reliance on laboratory confirmation before initiating response, and less effective local response [171, 177, 178]. Epidemic control more than 2 weeks post-onset carries a strong risk of epidemic propagation, particularly where the population is highly mobile. Our simple model simulations and sensitivity analyses suggest that with 3 seed cases, in 2 to 33% of scenarios such delays could result in clusters of 20 or more cases that would be difficult to contain. Comparatively, a field investigation and preliminary response to contain transmission done at the time of case presentation (~ 1 week) could potentially reduce the probability of reaching these epidemic sizes to 2 to 4% of scenarios. If larger outbreaks of 10 seed cases are detected, even within ~ 1 week, 95% of outbreaks could accumulate 20 or more cases, and thus would be difficult to contain.

Early detection and response are major aims of the *Ending Cholera* roadmap. There are two reasons to believe that policy and practice have somewhat narrowed the gap between detection and response. First, we found a global improvement in time to response that corroborates a previous analysis of improvements for detection of all-pathogen outbreaks in low- and middle-income countries from 1996 to 2014 [11]. This may be related to more attention and investment by governments and the GTFCC to the impacts of cholera epidemics in fragile states, given a decade of large and devastating cholera outbreaks in Haiti and across West and Central Africa, the Horn of Africa, and the Gulf of Aden [10]. Some countries appear to have documented improved capacity for detection and response, as shown in this analysis (e.g., Chad, Nepal, Somalia). This may be reflected by investments into strategies like the Joint External Evaluation process which have specified critical gaps for improvement [179–181].

Detailed case studies of cholera outbreaks provide practical observations on the mechanisms of surveillance, diagnosis, and response which can reduce delays. Early detection with high-quality epidemiological data has been augmented with the use of sentinel site surveillance at hospitals equipped with RDTs and trained and vigilant health workers in Kathmandu, Nepal [162]; community-based surveillance using existing community health worker or Red Cross volunteer networks to enable early warning of clusters in the community before patients appear at health facilities in Central African Republic and Haiti [71, 152, 182, 183]; and other event-based surveillance mechanisms, including phone hotlines and mobile phone fleets, to enable immediate notification of suspect events in public, private, and NGO clinics and in the community, as seen in Northern Nigeria and Cameroon [171, 184, 185]. Response should not be delayed by poor laboratory capacity. A potentially stronger role for health workers in local facilities exists in their use of enriched, high-specificity RDTs [186] and aligned probable case definitions to validate clusters of suspected cholera cases that can trigger an immediate investigation and response [162, 171]. This is directly applicable in remote districts and insecure areas where laboratory confirmation will be slow. Timely field investigation and preliminary response remains promising as most outbreak reports cited the use of an early warning alert system, with several examples of integrated investigation and response capacity. We consider that an integrated alert and at least a preliminary response to an outbreak within 1 week of onset should be possible in fragile settings [187]. However, despite the presence of EWARS in nearly 80% of the outbreaks examined, the median delay to response was 10 days. Where EWARS was used successfully to link early detection with a preliminary and robust response, for example in Afghanistan (2010–1), Nepal (2011–6), and Northern Nigeria (2018), a timely response was judged to be dependent on adequate and trained human resources (e.g., district-level rapid response teams or local health facility staff capable of multidisciplinary investigation and a generic response [63, 162]), and the ability to mount at least a preliminary response moving forward independent of laboratory confirmation [52, 75, 100, 101, 139, 161, 171]. For example, investigation and response were integrated in Afghanistan (2010) where a local NGO was trained rapidly to carry out a comprehensive community response, as they had more access to the area than health authorities in an insecure area [52]. In Chad (2017), investigation was carried out by the staff of a local health facility, who also initiated the preliminary community response [137].

It is important to consider the limitations inherent to a retrospective review of data from secondary sources. First, as no global registry of cholera outbreaks exists, we relied on the manual compilation of available situation reports and articles. The most comprehensive source, the WHO’s current compilation of annual cholera data, does not provide detailed information on outbreaks and misses non-reporting countries. The small annual numbers of outbreaks pre-2015 documented here may reflect the few global data sources available. As well, larger outbreaks are more likely to be detected, responded to, and therefore documented and included here. Second, the delays are estimates of reality; dates from situation reports are likely inaccurate to an unquantifiable degree as the exact dates of local investigation and response may be subjective and are documented infrequently. The identification of the primary case(s) depends on the depth of the field investigation, and with a multi-pathway pathogen like *Vibrio cholerae* that causes a range of disease severity, transmission chains may be missed. Fifty-one (67%) of the 76 outbreaks were missing the date of onset of symptoms for the primary case, which then had to be estimated, limiting accuracy. The dates of response were based on the judgment of the timing of the first transmission-reducing intervention and thus may represent variable intensity of response across outbreaks. Of note, the longest delays to response noted during outbreaks in Chad, Ethiopia, Somalia, and Uganda were related to the first viable response after an inadequate local response. To address these inconsistences, we sourced multiple reports per outbreak to triangulate the information and obtain a clear timeline, and excluded a large number of outbreaks where reports lacked detailed dates. Second, we note that while outbreaks are likely to occur during conflict, they are difficult to detect amidst violence where surveillance coverage is poor [19]. These outbreaks, with potentially high mortality, may have gone undetected, unless they occurred in urban areas and/or propagated to a point of being overwhelming, as in South Sudan in 2014 and Yemen in 2016 [4, 131]. Third, the simple branching process models used here for demonstrating early epidemic growth are for illustration purposes only. These models did not take into account key sources of uncertainty including initial susceptibility to infection (influenced by prior cholera infection or vaccination), heterogeneity in contact and transmission routes, depletion of susceptible persons, and the time-varying *R*_*t*_ value.

The documentation of the occurrence and features of cholera outbreaks is currently very heterogeneous. A real-time global database using standard outbreak event reports maintained by WHO regional offices to prospectively log data and metrics from outbreaks of cholera, as well as other epidemic-prone diseases, would yield superior accuracy for the annual evaluation of timeliness. WHO AFRO’s Weekly Bulletin on Outbreaks and Emergencies provides an existing template which can feed into such a global database [20]. WHO AFRO has used this tool to provide annual metrics of timeliness in outbreak response for epidemic-prone diseases from 2017 to 2019, demonstrating reduced time from symptom onset of the primary case to outbreak detection (defined as alerting national authorities) from 14 (IQR 6–37) days in 2017 to 4 (IQR 1–11) days in 2019 [188].

## Conclusions

Cholera epidemics will continue to appear unpredictably and cause serious morbidity and mortality in countries affected by armed conflict and fragility. Cholera surveillance and response is dependent on rethinking the timely detection, investigation, and response to primary cases at the local level. This includes reinforcing outbreak detection through event-based surveillance, consistent weekly reporting using standard case definitions, systematic use of enriched RDTs, and integrating early investigation with preliminary local response. These measures should increasingly underpin the detection and containment of emerging epidemics.

## Supplementary Information

**Additional file 1.** Countries investigated.

**Additional file 2.** Compilation of outbreaks by country, date of onset, delays (detection, investigation, response), signal, source, and description of investigation and response.

**Additional file 3.** Histograms of delays from symptom onset to (A) case presentation, (B) outbreak detection, (C) investigation, (D) response, and (E) confirmation.

**Additional file 4.** Overview of alternative models for the main analyses.

**Additional file 5.** Model parameters for additional delay analyses.

## Data Availability

The dataset supporting the conclusions of this article is available in the Github repository, [https://github.com/ruwanepi/Detection-analysis].

## Abbreviations

AIC: Akaike information criterion
AWD: Acute watery diarrhea
CI: Confidence interval
DEWS: Disease early warning system
EWARN: Early warning alert and response network
EWARS: Early warning alert and response system
FCAS: Fragile and conflict-affected situation
IQR: Interquartile range
GTFCC: Global Task Force for Cholera Control
NGO: Non-governmental organization
OCV: Oral cholera vaccination
*R*_E_: Effective reproduction number
RDT: Rapid diagnostic test
SD: Standard deviation
WASH: Water, sanitation, and hygiene
WHO: World Health Organization
