# Supplementary material for: Early detection of cholera epidemics to support control in fragile states: estimation of delays and potential epidemic sizes
Source: BMC Med. 2020 Dec 15;18:397. doi: 10.1186/s12916-020-01865-7 (PMC7737284; doi:10.1186/s12916-020-01865-7)
Supplement: Supplementary file 1 — Additional file 1. Countries investigated. [file 12916_2020_1865_MOESM1_ESM.docx]

**Additional file 1**

**Countries investigated**

| Fragile and conflict-affected states^1^ | Refugee-hosting country and/or borders one or more fragile state**^b^** |
| --- | --- |
| 1. Afghanistan 2. Angola 3. Burundi 4. Cambodia 5. Cameroon 6. Central African Republic 7. Chad 8. Congo, Dem. Rep. 9. Congo, Rep. 10. Côte d'Ivoire 11. Djibouti 12. Eritrea 13. Gambia, The 14. Guinea 15. Guinea-Bissau 16. Haiti 17. Iraq**^a^** 18. Lao, PDR 19. Liberia 20. Madagascar 21. Mali 22. Mozambique 23. Myanmar**^a^** 24. Nepal 25. Papua New Guinea 26. Sierra Leone 27. Somalia 28. South Sudan 29. Sudan 30. Syrian Arab Republic**^a^** 31. Tajikistan 32. Timor-Leste 33. Togo 34. Yemen, Rep. 35. Zimbabwe | 1. Benin 2. Ethiopia 3. Kenya 4. Niger 5. Nigeria 6. Tanzania 7. Uganda 8. Zambia |

^a^These fragile states^1^ did not appear on the list of the Global Task Force for Cholera Control list of cholera-affected countries^2^, but were included as they are known to have had cholera outbreaks from 2008-2019.

^b^These countries did not meet the criteria set for fragile states (appearing ≥2 times during 2008-2019 on the World Bank’s Harmonized List of Fragile Situations).^1^ However, these countries were included as they are considered cholera-affected using the Global Task Force for Cholera Control list of cholera-affected countries^2^ and are either (a) a refugee-hosting country and/or (b) are bordering a fragile or conflict-affected state.
