# Supplementary material for: Early detection of cholera epidemics to support control in fragile states: estimation of delays and potential epidemic sizes
Source: BMC Med. 2020 Dec 15;18:397. doi: 10.1186/s12916-020-01865-7 (PMC7737284; doi:10.1186/s12916-020-01865-7)
Supplement: Supplementary file 2 — Additional file 2. Compilation of outbreaks by country, date of onset, delays (detection, investigation, response), signal, source, and description of investigation and response. [file 12916_2020_1865_MOESM2_ESM.docx]

**Additional file 2**

**Outbreaks by country, date of onset, delays (detection, investigation, response), signal, source, and description of investigation and response**

| **ID** | **Country, area** | **Date**  **onset (DOS)** | **DOS to**  **detection**  **(days)** | **DOS to investigation (days)** | **DOS to response (days)** | **Signal** | **Source**  **(system)** | **Investigation and response** |
| --- | --- | --- | --- | --- | --- | --- | --- | --- |
| 1 | **Afghanistan**, Nawa district, Ghazni province[1, 2]   \|  \| \| --- \| | Aug 2010 | 6 | 6 | 6 | Cluster of 60 suspect cases in a remote and insecure district | Formal alert  (DEWS) | Through DEWS, an alert, investigation, and response was linked on the same day that the alert was issued, involving distribution of medical supplies and training of NGO workers with access to the site in investigation and control. The outbreak was reported as contained by Sept 2010. |
| 2 | **Afghanistan**, Giro district, Ghazni province[3, 4] | Apr 2011 | 21 | 21 | -- | Multiple clusters in a remote and insecure district | Formal alert  (DEWS) | Through DEWS, surveillance officers investigated rumours of multiple clusters and recorded 255 cases of AWD with dehydration. In parallel, they carried out a rapid response.  The first case was traced back two weeks before the outbreak was detected. The outbreak was reported as contained by Jul 2011. |
| 3 | **Angola**, Soyo City, Zaire Province[5-7] | Dec 2016 | 13 | -- | 32 | Multiple clusters in two towns in provinces bordering DRC | NR | The two clusters appeared close in time, and were judged to be linked to a larger outbreak in Kongo, Central Province, DRC. Linked transmission in Luanda was identified in January 2017. |
| 4 | **Angola**, Tchizo neighborhood, Cabinda Province[5, 7, 8] | Dec 2016 | 5 | -- | 17 | Multiple clusters in two towns in provinces bordering DRC | NR |  |
| 5 | **Angola**, Uige Town, Uige Province[5, 9, 10] | Dec 2017 | 5 | -- | 10 | Two suspect cases presenting to a health facility in urban area | NR | Two cases with travel history to Kimpangu, DRC presented close in time to a health facility. The outbreak was linked to the ongoing outbreak in DRC. |
| 6 | **Benin**, Littoral Department (outskirts of Cotonou)[11] | Jul 2008 | 5 | -- | 8 | Single suspect case presented to a health facility in an urban area | NR | The same health facility opened a CTC shortly after. |
| 7 | **Benin**, So-Tchanhoue village, So Ava Commune, Atlantique Department[12-14] | Feb 2016 | 5 | 8 | -- | Single suspect case presented to a health facility in a rural area | Formal alert  (routine surveillance with immediate notification) | The health facility alerted public health officials immediately, without waiting for the weekly epidemiological report. The response was driven by community health program and occurred rapidly. |
| 8 | **Benin**, Dekanme, So Ava commune, Atlantique Department[14-16] | Aug 2016 | 13 | 13 | 18 | Single suspect death presented at a health facility in a rural area | Formal alert  (routine surveillance through weekly report) | Routine surveillance was the source of the alert of a suspected cholera death, with one week's delay. Response was enabled by the pre-positioning of cholera kits in affected districts and a locally-driven strategy with community health workers. |
| 9 | **Burundi**, Rumonge, Bururi province[17, 18] | Jul 2011 | 7 | -- | 20 | Multiple suspect cases presented to a health facility in a town on Lake Tanganika | NR | The outbreak spread from Rumonge town to multiple provinces including Bujumbura Rural. CTCs were setup in response. |
| 10 | **Burundi**, Bujumbura town and Bujumbura Rural[19, 20] | Sept 2012 | 5 | -- | 9 | Multiple suspect cases were reported in an urban area and the wider province. | NR | A community-focused response was setup. |
| 11 | **Burundi**, District Sanitaire Nyanza-Lac, Makemba Province[21, 22] | Aug 2017 | 5 | -- | 5 | Single suspect case presented to a health facility in a rural area | Formal alert  (routine surveillance with immediate notification) | The case was immediately notified and transferred to a hospital for isolation and diagnostics. A CTC and community-based activities were setup. |
| 12 | **Burundi**, Rumonge, Bururi Province[23, 24] | Dec 2018 | 0 | 3 | 6 | Multiple suspect cases presented to a health facility in a single health district on Lake Tanganika. | NR | Three suspected cases presented to a health facility in a single health district within two days. A CTC was opened immediately. |
| 13 | **Cameroon**, Mora town, Mora and Maroua Districts[24] | Apr 2016 | 5 | 5 | 5 | Single cluster of 69 suspect cases reported after several cases presented to a hospital | Formal alert  (routine surveillance with immediate notification) | This was a false alert of a cholera signal (likely food poisoning) linked to a rapid investigation and response. A rumour of a large cluster was reported through the surveillance system. RDT tests that were positive were culture negative. Red Cross volunteers did household disinfection and community activities during the investigation period. |
| 14 | **Cameroon**, Boko health district, Littoral Department[25] | May 2016 | 5 | 5 | -- | Multiple suspect cases and one death among under 5 children | Informal alert  (rumour form community member) | This was a false alert of a cholera signal (likely rotavirus) linked to a rapid investigation and response. A rumour from a community leader involving suspect cases and a death among children closely in time was reported to public health authorities. On the same day, investigation and response was undertaken. |
| 15 | **Cameroon**, Guirviza health area and Doumo health area, Maya Oulo health zone[26] | May 2018 | 6 | 6 | 11 | Multiple suspect cases from two rural areas | Formal alert  (routine surveillance with immediate notification) | Cultures were taken on the same day, indicating an immediate alert to public health authorities. The investigation determined the cases were linked to travel to Nigeria, where there was an ongoing outbreak. Response included community activities, chlorination of water points, and training in case management. |
| 16 | **Central African Republic**, villages along the Ubangui River, 80 km south of Bangui[27-29] | Sept 2011 | 13 | 15 | 20 | Multiple suspect cases (and a death) in villages on the Ubangui River close to the capital | Informal alert  (rumour of a death reported from community) | Response required declaration by government and mobilization of international partners. Transmission to Bangui was registered within one month. |
| 17 | **Central African Republic**, Mourou-Fleuve village, Ndjoukou subprefecture[30, 31] | Jul 2016 | 5 | 5 | 14 | Multiple suspect cases in villages along a river, bordering DRC | Formal alert  (community-based surveillance) | Red Cross community volunteers detected and provided an immediate formal alert to the first cases. The outbreak was linked to another outbreak in DRC. |
| 18 | **Chad**, District Sanitaire Fianga, Mayo-Kebbi[32] | Jun 2011 | 5 | -- | 79 | Multiple suspect cases in a rural area | NR | 11-week delay in response due to poor mobilization of non-governmental support. |
| 19 | **Chad**, Marrena village, District Sanitaire Koukou, Sila Region[33-36] | Aug 2017 | 5 | 6 | 6 | Two suspect cases (resulting in deaths) among children presented to a health facility | Formal alert (immediate notification and investigation) | Two children presented and died upon admission with further cases from a remote village. Investigation on the same day (immediate formal notification) through the health facility found 50 cases and 13 deaths in Marena. Rapid response occurred the next day. |
| 20 | **Chad**, District Sanitaire Koukou, Sila Region[33, 34] | Aug 2017 | 5 | 5 | 11 | Multiple suspect cases (and deaths) presented to a health facility in a rural area bordering Sudan | Formal alert  (immediate notification and investigation) | Health facility reported multiple suspect cases and two deaths from villages near the border with Sudan. Response within 3 days occurred. |
| 21 | **Chad**, Angarana, District Sanitaire Koukou, Sila Region[33] | Aug 2017 | 5 | -- | 10 | NR | NR | NR |
| 22 | **Chad**, d'AmTiman, District Sanitaire d'AmTiman, Salamat Region[33] | Sept 2017 | 5 | -- | 7 | NR | NR | NR |
| 23 | **Chad**, Youe health district, Mayo Kebbi[37] | Jul 2019 | 5 | -- | 10 | Multiple suspect cases (including 1 death) from 1 neighbourhood presented to a health facility | NR | NR |
| 24 | **Congo**, Assemblee camp, Mbanou Island, Talangai Health District[38] | Aug 2016 | 6 | 7 | 7 | Multiple suspect cases were notified from a work camp | NR | Six suspected cases from a work camp among persons from DRC were notified. Investigation and response was rapid, and included disinfection of camp and health centre, chlorination activities, and community mobilization. By mid-Sept, no further cases were reported. |
| 25 | **Congo**, Likouala Department[39] | Mar 2018 | 5 | 19 | 19 | Multiple suspect cases from a village presented to a health facility | Informal alert  (immediate investigation and review of retrospective data) | Retrospective review of health facility records found 3 cases of AWD managed in previous week. A national rapid response team was deployed two weeks after notification to conduct investigations and a comprehensive response. |
| 26 | **Cote d'Ivoire**, Zimbabwe neighborhood, Abidjan[40, 41] | Sept 2014 | 5 | -- | 6 | Multiple suspect cases presented to health facility on an island close to the capital | NR | Eight Ghananian fisherman sought care on an island close to Abidjan. |
| 27 | **DRC**, Kinshasa (Camp Luka, Binza Meteo, Limete and Kintambo Health Zones)[42] | Nov 2017 | 5 | -- | 37 | Sudden increase in trend for AWD in urban area | Formal alert  (data analysis of case numbers) | Through IDSR, following intensive rains and flooding, a formal alert (data analysis of case numbers) showed an increase from <5 to >100 weekly suspect cases. A logistically-intensive and comprehensive CATI-like strategy was used for response. No further cases reported by late-Dec 2017. |
| 28 | **Ethiopia**, Moyale town and surrounding kebeles, Moyale Oromia and Moyale-Somali[43-46] | Nov 2015 | 1 | 51 | 51 | Large cluster of AWD cases in urban area | Formal alert  (data analysis of case numbers via EWARS) | In an area of displacement between Somalia and Ethiopia, WHO deployed a rapid response team to strengthen the outbreak response almost two months following detection of an AWD outbreak which had grown to 268 cases. A rapid decline in caseload was reported following the rapid response. |
| 29 | **Ethiopia**, Degah-ad Kabele, Danbal Woreda, Sitti Zone (near Jijiga city)[47] | Jun 2017 | 5 | 6 | 6 | Cluster of suspect cases near an urban area | Informal alert  (rumour of suspect cases via EWARS) | Rumour from community of 31 suspect cases in a community. Rapid response team in Jijiga City trained on case management and IPC; supported scale up of surveillance and community WASH measures. |
| 30 | **Guinea**, fishing village, Khounyi, Kabak Island, Forecariah[48] | Feb 2012 | 3 | 4 | -- | Cluster of suspect cases in a single fishing village | Formal alert  (immediate notification of a cluster) | Cluster of suspect cases in a single village alerted by a CHW carrying out vaccination as part of the sentinel surveillance system. |
| 31 | **Guinea-Bissau**, Tombali village, Tombali region[49-51] | Apr 2008 | 5 | -- | 20 | Cluster of suspect cases in a remote fishing village | NR | This outbreak was thought to be contained in Tombali village but linked transmission was detected in Bissau in Jul 2008. There was a notable delay in detection of the outbreak of 2 weeks. |
| 32 | **Haiti**, Mirebalais, Artibonite Department[52, 53] | Oct 2010 | 7 | 9 | 9 | Sudden increase in trend for AWD and dehydration in urban areas | Formal alert  (data analysis of case numbers) | Ministry of Health notified of unusually high peaks of AWD and dehydration cases (>1,000 cases), and some deaths from Centre and Artibonite departments |
| 33 | **Iraq**, Al-Auzeir town, Maysan Governorate[54-57] | Aug 2008 | 12 | -- | 31 | Single suspect case (death) among a child in a town, followed by other case reports | Formal alert  (data analysis of case numbers through national diarrhoea surveillance) | A child died of suspected cholera. Initial laboratory tests were inconclusive. A delay in laboratory confirmation and notification to the central authority delayed comprehensive outbreak response activities. |
| 34 | **Iraq**, Najaf City, Baghdad Governorate[58] | Aug 2015 | 9 | 14 | 15 | Sudden increase in trend for AWD in urban area | Formal alert  (data analysis of case numbers) | Single suspect case detected at Najaf Hospital but routine analysis of diarrheal cases from district served as alert of increase in diarrheal cases and earlier cases traced back to a week before. Field investigation and a preliminary local response was rapidly conducted. |
| 35 | **Kenya,** Kakuma refugee camp, Turkana District[59] | Sept 2009 | 5 | -- | 18 | Sudden increase in trend for AWD in refugee camp | Formal alert  (data analysis of case numbers) | Incidence of AWD among camp residents increased sharply in Sept 2009, at same time of first clinical encounters with suspect cases. The context was a larger outbreak in Turkana District since Aug 2009. Large-scale response started in Oct 2009. |
| 36 | **Kenya,** Hagadera refugee camp, Dadaab complex, Garissa District[60] | Mar 2019 | 5 | -- | 7 | NR | NR | The delay between reporting of the index case and notification was due to surveillance challenges. A CTC was setup shortly after the first case reports. |
| 37 | **Liberia**, Maryland and Grand Kru counties[61, 62] | Nov 2007 | 7 | -- | 23 | Sudden increase in AWD (and deaths) reported, mostly from one hospital in an urban area | NR | Suspect cases detected between early Dec 2007 and Jan 2008. The response (development of an CTU at the hospital) occurred 2 weeks after presentation of the first cases. |
| 38 | **Liberia**, Tapitta district, Nimba County[63, 64] | Mar 2017 | 5 | 7 | 7 | Single suspect case died en-route to health facility in urban area | Formal alert  (immediate notification through IDSR) | The outbreak was detected within 2 days of the first case (a death among an adolescent) presenting to a health facility. A rapid response team was dispatched on the same day. The outbreak was reported as contained within the same week. |
| 39 | **Mali,** Wabaria District, Gao region[65] | Jun 2012 | 5 | 7 | 7 | Single cluster reported in a rural village | NR | Sudden appearance of 32 suspected cases. Response included implementation of a CTC by ICRC, who was already present, and use of Red Cross volunteers for community mobilization. |
| 40 | **Mozambique** (Cyclone Idai), Beira city and districts of Nhamatanda, Dondo, Buzi, Sofola Province[66-68] | Mar 2019 | 5 | -- | 12 | NR | Formal alert  (immediate notification using EWARS) | Vigilance and rapid detection of an outbreak following Cyclone Idai, and rapid growth, followed by rapid response involving OCV. |
| 41 | **Mozambique** (Cyclone Kenneth), Pemba city and Mecufi district[69] | Apr 2019 | 5 | -- | 9 | Multiple clusters reported in two areas | Formal alert  (immediate notification using EWARS) | Vigilance for cholera due to prior experience with Cyclone Idai. The first suspect case observed on 27 April 2019 and outbreak declared after multiple clusters in Pemba city and Mecúfi district. Rapid response within four days of detecting the first case. |
| 42 | **Nepal,** Tilathi VDC[70] | Oct 2011 | 5 | 6 | 6 | Single cluster (with deaths) in an urban area | Formal alert  (immediate notification via early warning function) | District public health authorities notified of a cluster in an urban area. The next day, the outbreak control team investigated and responded. |
| 43 | **Nepal,** Kathmandu Valley[71, 72] | Jun 2016 | 5 | -- | 9 | Single suspect case presented to a health facility in an urban area | Formal alert  (immediate notification using sentinel surveillance and RDTs) | Sentinel site surveillance of AWD using RDT was used after the earthquake in Nepal. Rapid response team respond to suspect cases. |
| 44 | **Nepal,** Gaidataar, Chandranigahpur VDC-3 and 4[73] | Apr 2017 | 5 | 6 | 6 | Sudden increase in trend for AWD in urban area | Formal alert  (immediate notification using EWARS) | Via EWARS, increase of AWD detected mid-Apr 2017. On same evening, two suspect cases admitted to hospital. The next day, a rapid response team sent to investigate and control. |
| 45 | **Niger**, Bella village, Dosso Health District[74, 75] | Oct 2016 | 5 | 11 | -- | Single cluster (with 9 deaths) in a rural village | Informal alert  (rumour of suspect cases and deaths) | A rumour of gastroenteritis cases from one village, including 9 community and health facility deaths. An MoH, WHO, and UNICEF investigation team was sent within a week. |
| 46 | **Niger**, Madarounfa, Maradi District[76] | Jun 2018 | 7 | 7 | -- | Single cluster (one death) among one family presenting to a health facility in a rural village | Informal alert  (rumour of suspect cases and deaths) | A suspected cluster was notified among a family of cases admitted to a health facility. Investigation found travel history to Nigeria during exposure period. |
| 47 | **Nigeria**, Gomani settlement, Kundu ward of Kwali LGA, Federal Capital Territory[77] | Oct 2014 | 13 | 13 | 13 | Single cluster presenting to a health facility | Formal alert  (immediate notification via early warning function) | Health facility reported to surveillance officer an increase in suspect cases in a single village. The investigation traced back an index case to 2 weeks earlier that the date of detection. The outbreak was reported as contained within a week. |
| 48 | **Nigeria**, Muna Garage IDP camp, Jere LGA, Borno State[78-80] | Aug 2017 | 2 | 2 | 9 | Single suspect case of AWD in a refugee camp | Formal alert  (immediate notification using EWARS) | First case was notified by MSF via phone call to EWARS, triggering an investigation on the same day. The delay in laboratory confirmation (declared negative at local labpratpry and positive by national laboratory weeks later) delayed a comprehensive response. |
| 49 | **Nigeria**, Doro Ward, Kukawa LGA, Borno State[81] | Feb 2018 | 5 | 13 | 14 | Single cluster in an urban area | NR | NR |
| 50 | **Pakistan,** Mingora, Swat Valley[82-84] | Jul 2010 | 5 | -- | 5 | Gradual increase in trend for AWD across flood-affected area | Formal alert  (data analysis of case numbers) | One case was confirmed in Mingora, Swat Valley. Suspect cases were detected, and the response assumed cholera. A trend in confirmed cases was identified. |
| 51 | **Pakistan,** Amarpura, Rawalpindi[85] | Jul 2017 | 0 | 3 | -- | Single case confirmed among a paediatric patient already admitted in hospital | Formal alert  (immediate notification) | Health facility notified of a confirmed case among a pediatric patient already admitted to hospital. Following an investigation, no further cases were reported. Response included chlorination of water tanks in households and community, isolation of cases, and active case finding. |
| 52 | **Papua New Guinea,** Nambariwa, Morobe province[86, 87] | Jul 2009 | 15 | 22 | 22 | Single cluster in a remote village | Informal alert  (rumour from community) | A physician visiting family reported an outbreak of AWD associated with death of his father and 4 persons from two villages. Response included active case-finding, isolation, and improvement of WASH. |
| 53 | **Sierra Leone,** Island of Yeliboya, Kambia[48, 88] | Jan 2012 | 5 | 8 | 8 | Single cluster of AWD in an island | Formal alert  (immediate notification) | Physician notified central public health authorities of increase in AWD trend. Investigation occurred within two days of notification. Confirmation took one month (at laboratory in Burkina Faso). |
| 54 | **Somalia,** Luuq and Belet Xawa, Gedo Region[89] | Nov 2008 | 5 | 66 | -- | NR | NR | Outbreak remained undetected over 12 weeks causing relatively high mortality and morbidity. Long time lag between submission of samples and confirmation, and a comprehensive response. |
| 55 | **Somalia,** Belet Xaawo (Belet Hawa)[90] | Apr 2016 | 5 | -- | 14 | Single cluster at a health facility in urban area | Formal alert  (immediate notification via early warning function) | A district hospital alerted other health facilities to an increase in AWD trend when the hospital admitted the first suspected case. Response included community prevention measures. |
| 56 | **Somalia,** Beletweyne district, Hiraan region[91, 92] | Dec 2018 | 5 | 5 | 19 | Sudden increase in trend for AWD in urban area | Formal alert  (data analysis via EWARS) | Increased trend in AWD was notified via EWARS and confirmed rapidly, Response including opening a CTC and training CHWs on community case management of cholera. |
| 57 | **South Sudan,** Yei town[93-96] | Feb 2008 | 29 | 34 | 34 | Multiple suspect cases presenting to a health facility in a remote and insecure area | NR | The response appeared delayed for 3 weeks due to a need for external non-governmental support (Medair provided support for the response). |
| 58 | **South Sudan,** Juba 3 IDP camp, Gudelle 2[97-99] | Apr 2014 | 6 | 6 | 22 | Single suspect case presenting to a health facility in a camp | Formal alert  (immediate notification via EWARS) | A single suspect case was notified by MSF on the day of presentation. Confirmation was achieved in a week, and a comprehensive response followed. OCV had been deployed preventatively in Juba 2 months before. |
| 59 | **South Sudan,** 50 villages in seven payams of Juba County[94, 100] | May 2015 | 8 | 9 | 9 | Multiple suspect cases presenting to health facility in a camp | Formal alert  (immediate notification via EWARS) | Investigation of the initial cases occurred the day following case presentation. |
| 60 | **South Sudan**, 11 villages in 6 payams in Juba[94, 101] | Jun 2016 | 0 | 15 | 15 | Multiple suspect cases reported from multiple locations | Formal alert  (immediate notification via EWARS and RDT testing) | RDT testing used for alerts issued through EWARS. Cases first observed in host community, then among IDPs. |
| 61 | **South Sudan,** Tonj East and Tonj North Counties, Warrap State[102] | May 2017 | 11 | 12 | 15 | Single cluster in a rural payam | Formal alert  (immediate notification via EWARS) | District-level public health authorities notified of a cluster in a rural payam. WHO supported a rapid response |
| 62 | **Sudan,** Ganees Shareg area of El Roseires locality, Blue Nile State[103-106] | Aug 2019 | 5 | -- | 5 | Single cluster (including 1 death) at a single hospital in an urban area | Formal alert  (immediate notification via early warning function) | El Roseires hospital notified of 5 suspect cases (and a death). The comprehensive response started <2 weeks later. |
| 63 | **Syria,** Aleppo Governorate, Eastern Rural[107, 108] | Oct 2015 | 2 | 2 | -- | Single suspect case (leading to death) in a rural and insecure area | Formal alert  (immediate notification via EWARN and RDT testing) | Via EWARN, a five year old child with AWD and dehydration was reported after being admitted to hospital (and death). Case was RDT+ but died before stool sample could be taken. Field investigation showed no symptomatic persons among household members. No formal response. |
| 64 | **Syria,** Zogra camp, near Jarabulus City, Aleppo[109, 110] | Oct 2017 | 5 | 5 | -- | Single suspect case in a camp in an insecure area | Formal alert  (immediate notification via EWARN and RDT testing) | This suspect case was likely a false alert. Via EWARN, alert of a suspected case among an infant of 4 months with AWD. Child was treatment and discharged; further investigation of the case showed that symptoms did not fit the case definition for cholera. No formal investigation in community or response. |
| 65 | **Tanzania,** Dar es Salaam (Kinondoni district)[111, 112] | Aug 2015 | 5 | 5 | 8 | Single cluster (and death) primarily among one family in an urban area | Formal alert (immediate notification) | Unknown source notified MoH of a suspect case of AWD with severe dehydration. Four family members identified as suspect cases with 1 death. Response was targeted to neighbourhood of cases. |
| 66 | **Tanzania,** Kigoma on Lake Tanganyika, in the nearby villages of Kagunga and Nyarugusu[113, 114] | May 2015 | 5 | 7 | 9 | Single cluster of two suspect cases (leading to death) in a refugee camp | Formal alert (immediate notification) | Two adults died, with symptoms of diarrhea and vomiting. AWD cases were considered suspect cholera, though cultures were initially testing negative for cholera. Active case-finding initiated and UNHCR sent treatment supplies. |
| 67 | **Uganda,** psychiatric hospital, Kampala[115] | Oct 2008 | 2 | 2 | 3 | Single cluster (and death) in a psychiatric hospital in an urban area | Formal alert (immediate notification) | Cluster of patients in same ward developed AWD and died rapidly. The hospital team suspected cholera and initiated control measures immediately including case management and antibiotic chemoprophylaxis. |
| 68 | **Uganda,** Bwere sub-county, Kasese District[116] | Mar 2015 | 22 | 84 | 84 | Single suspect case among a child presented to a heath facility | Formal alert (immediate notification with RDT) | The district health officer notified MoH of a RDT-positive suspect case with travel history to DRC. Despite local efforts, the outbreak continued to expand. Two months later, a comprehensive response was organized by the MoH. The investigation found suspect cases traced back to a month before outbreak detection. The outbreak lasted for 6 weeks with 183 suspect cases. |
| 69 | **Uganda,** Katwe village, Kasese District[117] | Jun 2015 | 4 | 6 | 6 | Single suspect case among a fisherman who presented to a hospital in a fishing village | Formal alert (immediate notification with RDT) | Rapid control was initiated with community hygiene measures, household chlorination and investigation. Outbreak reported contained within one month, with 61 suspect cases. |
| 70 | **Uganda,** Kyangwali refugee settlements, Hoima district[118] | Feb 2018 | 0 | -- | 6 | Single cluster (with deaths) among refugees at a reception centre | Informal alert | Cluster of suspect cases (and deaths) alerted from a refugee reception centre in a short time period. Outbreak coordination set up rapidly. |
| 71 | **Yemen,** Alshat and Ras Alara districts, Lahj Governorate[119] | Jun 2010 | 5 | -- | 10 | NR | NR | Outbreak started at the end of Jun 2010, with a large number (n=300) of suspect cases including 4 deaths. Comprehensive control measures including case management, we chlorination initiated by the MoH. |
| 72 | **Yemen,** Al-Razi hospital, Shokra Hospital, Abyan Governorate[120-122] | Apr 2011 | 5 | 60 | 70 | Sudden increase in trend for AWD in urban area | Formal alert  (data analysis via routine surveillance) | During an armed conflict, routine surveillance system detected a large increase in diarrheal cases in Abyan governorate. At detection, the outbreak was large (n=343 suspect cases). WHO supported a comprehensive response within 2 months. |
| 73 | **Yemen,** Sana'a (Al-Nasr neighbourhood of the Sho'ob district)[123, 124] | Oct 2016 | 5 | 6 | 14 | Multiple clusters detected in Sana’a City though presentation to a hospital | Formal alert (immediate notification with RDT) | MoH declared 8 confirmed cases admitted to a hospital in Sana’a, possibly from the same family. A WHO-supported rapid response team investigated and tested the cases. Shortly after, multiple villages in Al-Beyda district report suspect cases including deaths. Confirmation occurs a few days later. |
| 74 | **Zambia,** Chipata sub-district, Lusaka, and spreading to Kanyama sub-district around Oct 9, 2017[125-128] | Oct 2017 | 8 | -- | 17 | Single cluster of two suspect cases presented to a health facility in an urban area | NR | Two patients had presented at an urban clinic with symptoms. Coordination efforts occur shortly after. |
| 75 | **Zambia,** Nsumbu, Nsama District, Northern Province[129] | Mar 2019 | 0 | 0 | 0 | Multiple clusters (and deaths) from multiple villages in a rural area reported | NR | Index case among a child presented to a health facility and was isolated, but left prematurely. A traditional healer who later saw the child referred the child back to the health facility (died in transit). Multiple cases from the same household and a neighbouring village presented and were isolated. Outbreak was reported as contained. |
| 76 | **Zambia,** Mpulungu District, Northern Province[128] | Apr 2019 | 5 | 5 | -- | Single case presented to health facility in urban area. | NR | Index case among a child presented to the health facility with AWD, vomiting, and dehydration and deteriorated. Patient isolated and public health authorities alerted. Culture-positive results returned with delay a month later. |
| 77 | **Zimbabwe,** St Mary's and Zengeza sections of Chitungwiza city, Harare Province[130-132] | Aug 2008 | 3 | -- | 15 | NR | NR | MoH and MSF rapidly set up two CTCs in a hospital and in the community. Outbreak was reported as contained, but two months after this outbreak, a second wave of cases was reported across Harare suburbs and eventually in every province in the country. |
| 78 | **Zimbabwe,** Harare[133-135] | Sept 2018 | 4 | 4 | 5 | Single, large cluster (with deaths) presented to a hospital in an urban area | Formal alert (immediate notification with RDT) | At detection, a large cluster of 25 suspect cases were admitted to hospital. A concurrent typhoid outbreak in Harare stretched response capacity. |
| 79 | **Zimbabwe,** Chegutu municipality, Mashonland West Province[136-138] | Jan 2018 | 13 | 16 | 16 | Single cluster (with deaths) presented to a health facility in an urban area | Formal alert  (immediate notification via early warning function) | A small cluster of 5 suspect cases admitted to hospital, and 3/5 cases died within hours of admission. On the same day, public health authorities were notified of a suspected outbreak and investigated. A case in a woman who died at home after seeking treatment at a private clinic was retrospectively identified. Her funeral provided the epidemiological link to the current caseload. A rapid response team was sent to the Chegutu area to carry out a comprehensive response. The outbreak was reported as contained. |

References

1. Government of Afghanistan, WHO: Afghanistan: Cholera outbreak under control, say MoPH, WHO. Government of Afghanistan and World Health organization. 2010(July 7, 2020): <https://reliefweb.int/report/afghanistan/afghanistan-cholera-outbreak-under-control-say-moph-who>. Accessed August 28, 2010.

2. WHO EMRO: Suspected cholera in Afghanistan. WHO Regional Office for the Mediterranean. 2010, **3**(18): <http://applications.emro.who.int/dsaf/epi/2010/Epi_Monitor_2010_3_38.pdf?ua=1>. Accessed November 16, 2020.

3. WHO EMRO: Cholera in Afghanistan. WHO Regional Office for the Eastern Mediterranean. 2011, **4**(18 & 19): <http://applications.emro.who.int/dsaf/epi/2011/Epi_Monitor_2011_4_18-19.pdf?ua=1>. Accessed November 16, 2020.

4. WHO EMRO: Suspected cholera outbreaks in Afghanistan. WHO Regional Office for the Eastern Mediterranean. 2011, **4**(26 & 27): <http://applications.emro.who.int/dsaf/epi/2011/Epi_Monitor_2011_4_26_27.pdf?ua=1>. Accessed November 16, 2020.

5. Moore S, Dunoyer J, Sudre B, Valingot C, Rebaudet S, Piarroux R: Epidemiological study of cholera hotspots and epidemiological basins in East and Southern Africa. Prospective and Cooperation. 2018: <https://plateformecholera.info/attachments/article/639/Cholera%20epidemiology%20in%20South%20Sudan_UNICEF_April%202018_FINAL.pdf>. Accessed November 16, 2020.

6. Joint Cholera Initiative for Southern Africa (JCISA): Sub-regional up-date on Cholera: Week 4. Joint Cholera Initiative for Southern Africa (JCISA. 2017: <https://reliefweb.int/sites/reliefweb.int/files/resources/jcisa_bulletin_8_week_4jan2017.pdf>. Accessed November 12, 2020.

7. Joint Cholera Initiative for Southern Africa (JCISA): Sub-regional up-date on Cholera; Week 8. Joint Cholera Initiative for Southern Africa (JCISA),. 2017: <https://reliefweb.int/sites/reliefweb.int/files/resources/jcisa_bulletin_10_week_8feb2017.pdf>. Accessed November 12, 2020.

8. ProMED-mail.: Cholera, diarrhea & dysentery update (02): Africa. ProMED-mail. 2017, **13 Jan: 20170113.4751889**: <http://www.promedmail.org>. Accessed November 12, 2020.

9. WHO AFRO: Cholera in Angola. WHO Regional Office for Africa. 2018, **Week 3: 13-19 January 2018**: <https://apps.who.int/iris/bitstream/handle/10665/259885/OEW03-1319012018.pdf;jsessionid=90D67FBFDC659F22E19940CA3380679E?sequence=1>. Accessed November 16, 2020.

10. WHO AFRO: Cholera in Angola. WHO Regional Office for Africa. 2018, **Week 6: 3-9 February 2018**: <https://apps.who.int/iris/bitstream/handle/10665/260157/OEW6-030922018.pdf?sequence=1>. Accessed November 16, 2020.

11. Gbary AR, Dossou JP, Sossou RA, Mongbo V, Massougbodji A: **[Epidemiologic and medico-clinical aspects of the cholera outbreak in the Littoral department of Benin in 2008]**. *Med Trop (Mars)* 2011, **71**(2):157-161.

12. UNICEF: Cholera Outbreaks in Central and West Africa: 2016 Regional Update. UNICEF West and Central Africa. 2016, **Week 04**: <https://reliefweb.int/sites/reliefweb.int/files/resources/WCA_Cholera_Update_W4.pdf>. Accessed November 16, 2020.

13. UNICEF: Cholera outbreaks in the West and Central Africa: 2016 Regional Update. UNICEF West and Central Africa. 2016, **Week 6**: <https://reliefweb.int/sites/reliefweb.int/files/resources/snapshot_wca_unicef_cholera_update_w6.pdf>. Accessed November 16, 2020.

14. Ministere de la sante de la Repulique du Benin: Plan D'Endiguement du Cholera, 2017-2021. 2017: <http://plateformecholera.info/attachments/article/430/Plan%20d'Endiguement%20Cholera%20du%20Benin.pdf>. Accessed November 16, 2020.

15. UNICEF: Cholera Outbreaks in Central and West Africa: 2016 Regional Update. UNICEF West and Central Africa Regional Office. 2016, **Week 10**: <https://reliefweb.int/sites/reliefweb.int/files/resources/WCA_Cholera_Update_W10.pdf>. Accessed November 16, 2020.

16. UNICEF: Benin: Update Choléra. 2016: <https://reliefweb.int/sites/reliefweb.int/files/resources/UNICEF%20Be%C3%ACnin%20Cholera%20Update%2022%20September%202016.pdf>. Accessed November 16, 2020.

17. Unknown: A Dozen killed in cholera outbreak. The New Humanitarian. 2011: <https://www.thenewhumanitarian.org/news/2011/09/14/dozen-killed-cholera-outbreak>. Accessed November 16, 2020.

18. International Federation of the Red Cross and Red Crescent: Disaster relief emergency fund (DREF). Burundi: Cholera. International Federation of the Red Cross and Red Crescent. 2011(GLIDE n° EP-2011-000126-BDI): <https://reliefweb.int/sites/reliefweb.int/files/resources/Full_Report_2277.pdf>. Accessed November 16, 2020.

19. International Federation of the Red Cross and Red Crescent: Disaster relief emergency fund (DREF) final report, Burundi: Cholera, DREF operation n° MDRBI009. International Federation of the Red Cross and Red Crescent. 2013(GLIDE n° EP-2012-000187-BDI): <https://reliefweb.int/sites/reliefweb.int/files/resources/Burundi%20Cholera%20DREF%20Operation%20Final%20Report.pdf>. Accessed November 16, 2020.

20. Croix-Rouge du Burundi: La Croix-Rouge du Burundi dans la luttre contre l'épidémie de choléra. Croix-Rouge du Burundi. 2012: <https://reliefweb.int/report/burundi/la-croix-rouge-du-burundi-dans-la-lutte-contre-l%E2%80%99%C3%A9pid%C3%A9mie-de-chol%C3%A9ra>. Accessed November 16, 2020.

21. WHO AFRO: Cholera, Burundi. WHO Regional Office for Africa. 2017, **Week 34: 19-25 August 2017**: <https://apps.who.int/iris/bitstream/handle/10665/258794/OEW34-192582017.pdf;jsessionid=93BF65989F3FC960D06F2813029628B7?sequence=1>. Accessed November 16, 2020.

22. WHO AFRO: Flambee des cas de cholera au Burundi. WHO Regional Office for Africa. 2017: <https://reliefweb.int/sites/reliefweb.int/files/resources/whe-burundi_cholera_update_002_21082017_0.pdf>. Accessed November 16, 2020.

23. WHO AFRO: Cholera in Burundi. WHO Regional Office for Africa. 2019, **Week 1: 29 December 2018-4 January 2019**: <https://apps.who.int/iris/bitstream/handle/10665/278952/OEW01-29122018-04012019.pdf?sequence=1&isAllowed=y>. Accessed November 16, 2020.

24. Government of Burundi, WHO: Rapport de situation n°01 sur la flambee de cholera au Burundi. WHO. 2018: <https://reliefweb.int/sites/reliefweb.int/files/resources/30122018_OMS%20Burundi%20Flamb%C3%A9e%20CHOLERA_RAPSIT.pdf>. Accessed November 16, 2020.

25. UNICEF: Cholera alert lifted in Mora, Mayo-Sava Department, Extreme North Cameroon. UNICEF’s West and Central Africa Regional Office. 2016, **Week 18**: <https://reliefweb.int/sites/reliefweb.int/files/resources/WCA_Cholera_Update_W18.pdf>. Accessed November 16, 2020.

26. UNICEF: Early warning and early actions in Cameroon, Littoral Region. UNICEF West and Central Africa Regional Office. 2016, **Week 26**: <https://reliefweb.int/sites/reliefweb.int/files/resources/WCA_Cholera_Update_W26_-_V1.pdf>. Accessed November 16, 2020.

27. International Federation of the Red Cross and Red Crescent: Disaster Relief Emergency Fund (DREF) final report, Central African Republic: Cholera outbreak, DREF operation n° MDRCF009. International Federation of the Red Cross and Red Crescent. 2012(GLIDE n° EP-2011-000153-CAF): <https://reliefweb.int/sites/reliefweb.int/files/resources/MDRCF009finrep.pdf>. Accessed November 16, 2020.

28. Larsen DC: UNICEF responds to new cholera outbreak claiming lives in Central African Republic. UNICEF 2011: <https://www.unicef.org/media/media_60000.html>. Accessed November 16, 2020.

29. Penguele A., Djeintote M., Balekouzou A., Tembeti J., Feilema P., Kazambu D., D. D: Cholera Outbreak Investigation in the Central African Republic, October – November 2011. Unknown. 2011: <https://www.cdcfoundation.org/sites/default/files/upload/image/2011CholeraOutbreakReport.pdf>. Accessed November 16, 2020.

30. UNICEF: Highlights: Central African Republic. UNICEF West and Central Africa Regional Office. 2016, **Week 30**: <https://reliefweb.int/sites/reliefweb.int/files/resources/WCA_Cholera_Update_W30.pdf>. Accessed November 16, 2020.

31. International Federation of the Red Cross and Red Crescent: Emergency Plan of Action (EPoA), Central Africa Republic: Cholera Epidemic Outbreak. International Federation of the Red Cross and Red Crescent. 2016: <https://reliefweb.int/sites/reliefweb.int/files/resources/MDRCF021.pdf>. Accessed November 12, 2020.

32. Dunoyer J, Sudre B: Rapport de capitalisation au sujet de l'épidémie de choléra au Tchad, 2010. Ministere de la Sante Publique. 2011: <https://reliefweb.int/sites/reliefweb.int/files/resources/rapport-de-capitalisation-epid%C3%A9mie-de-chol%C3%A9ra-au-tchad-2010-acf.pdf>. Accessed November 16, 2020.

33. Monnard G: Reponse à l'épidémie de cholera au Sila et au Salamat, Tchad, 2017: rapport de synthese. UNICEF Chad. 2018: <https://plateformecholera.info/attachments/article/556/UNICEF%202018%20Rapport%20synth%C3%A8se%20r%C3%A9ponse%20%C3%A9pid%C3%A9mie%20chol%C3%A9ra%20Sila%20-%20Salamat%202017%20FINAL.pdf>. Accessed November 16, 2020.

34. WHO AFRO: Cholera, Chad. WHO Regional Office for Africa. 2017, **Week 34: 19-25 August 2017**: <http://apps.who.int/iris/bitstream/10665/258794/1/OEW34-192582017.pdf>. Accessed November 16, 2020.

35. UNICEF: Alert of suspected cholera outbreak - Eastern Chad - Sila Region - Koukou Angarana (close of Goz Beida). UNICEF West and Central Africa Regional Office. 2017, **Week 32**: <https://reliefweb.int/sites/reliefweb.int/files/resources/WCA_Cholera_Update_W32_-_V2.pdf>. Accessed November 16, 2020.

36. WHO Hub/Est (Abéché): Situation des cas de cholera dans la delegation sanitaire regionale du Sila. WHO Chad. 2017: <https://reliefweb.int/sites/reliefweb.int/files/resources/sitrep_cholera_sila_17_sept_2017.pdf>. Accessed November 16, 2020.

37. WHO AFRO: Cholera, Chad. WHO Regional Office for Africa. 2019, **Week 30: 22-28 July 2019**: <https://apps.who.int/iris/bitstream/handle/10665/326098/OEW30-2228072019.pdf>. Accessed November 16, 2020.

38. UNICEF: Highlights: Focus on cholera alert in Congo Brazzaville (22/09/2016). UNICEF West and Central Africa Regional Office. 2016, **Week 36**: <https://reliefweb.int/sites/reliefweb.int/files/resources/WCA_Cholera_Update_W36.pdf>. Accessed November 16, 2020.

39. WHO AFRO: Cholera in Congo. WHO Regional Office for Africa. 2018, **Week 16: 14-20 April 2018**: <https://apps.who.int/iris/bitstream/handle/10665/272386/OEW16-142042018.pdf>. Accessed November 16, 2020.

40. UNICEF: Ivory Coast: Cholera and fishermen movement along the coast. UNICEF West and Central Africa Regional Office. 2014, **Week 2**: <https://reliefweb.int/sites/reliefweb.int/files/resources/Cholera_W2_2015%20West%20and%20Central%20Africa.pdf>. Accessed November 16, 2020.

41. UNICEF: Cote d'Ivoire: Confirmation of a cholera outbreak in Abidjan. UNICEF West and Central Africa Regional Office. 2014, **Week 41**: <https://reliefweb.int/sites/reliefweb.int/files/resources/Cholera%20regional%20update_W41_2014%20WCA.pdf>. Accessed November 16, 2020.

42. Bompangue D, Moore S, Taty N, Impouma B, Sudre B, Manda R, Balde T, Mboussou F, Vandevelde T: **Description of the targeted water supply and hygiene response strategy implemented during the cholera outbreak of 2017-2018 in Kinshasa, DRC**. *BMC Infect Dis* 2020, **20**(1):226.

43. WHO AFRO: Ethiopia - Response for Acute Watery Diarrhea outbreak in Moyale town. WHO Regional Office for Africa. 2016: <https://www.afro.who.int/news/ethiopia-response-acute-watery-diarrhea-outbreak-moyale-town>. Accessed November 16, 2020.

44. Tull K: **Humanitarian interventions in Ethiopia responding to acute watery diarrhoea**. In: *Knowledge, evidence and learning for development (K4D).* Brighton, UK: Institute of Development Studies; 2018.

45. UNICEF: Ethiopia: Humanitarian Situation Report. UNICEF. 2015, **SitRep #7**: <https://reliefweb.int/sites/reliefweb.int/files/resources/UNICEF%20Ethiopia%20Humanitarian%20Sitrep%20No.%207%2C%20November%20-%20December%202015.pdf>. Accessed November 16, 2020.

46. Ethiopian Public Health Institute: Suspected AWD cases. Ethiopian Public Health Institute. 2016, **2**(10): <https://www.ephi.gov.et/images/pictures/Weekly-Epidemiological-Bulletin-2016-10%20(1).pdf>. Accessed November 16, 2020.

47. WHO AFRO: WHO responds to new acute watery diarrhea outbreak rumour in a remote zone in Somali region of Ethiopia. WHO Regional Office for Africa. 2017: <https://www.afro.who.int/news/who-responds-new-acute-watery-diarrhea-outbreak-rumour-remote-zone-somali-region-ethiopia>. Accessed November 16, 2020.

48. Dunoyer J, Sudre B, Rebolledo J, Cottavoz P, Rossi M: **Le choléra transfrontalier en Sierra Leone et Guinée en 2012 et les stratégies d'intervention associées**. In*.* Paris, France; 2013.

49. Luquero FJ, Banga CN, Remartínez D, Palma PP, Baron E, Grais RF: **Cholera epidemic in Guinea-Bissau (2008): the importance of "place"**. *PloS one* 2011, **6**(5):e19005.

50. Unknown: Guinea-Bissau: Cholera epidemic reaches capital, Bissau. The New Humanitarian. 2008: <https://reliefweb.int/report/guinea-bissau/guinea-bissau-cholera-epidemic-reaches-capital-bissau>. Accessed November 16, 2020.

51. Unknown: Guinea-Bissau: WHO confirms cholera outbreak. The New Humanitarian. 2008: <https://reliefweb.int/report/guinea-bissau/guinea-bissau-who-confirms-cholera-outbreak>. Accessed November 16, 2020.

52. Centers for Disease CaP: **Update: cholera outbreak --- Haiti, 2010**. *MMWR Morb Mortal Wkly Rep* 2010, **59**(45):1473-1479.

53. Ivers LC, Walton DA: **The "first" case of cholera in Haiti: lessons for global health**. *Am J Trop Med Hyg* 2012, **86**(1):36-38.

54. WHO EMRO: Cholera in Iraq. WHO Regional Office for the Mediterranean 2008, **1**(36): <https://applications.emro.who.int/dsaf/epi/2008/Epi_Monitor_2008_1_36.pdf?ua=1&ua=1>. Accessed November 16, 2020.

55. WHO: **Cholera, Iraq**. *Weekly Epidemiological Record* 2008, **39**(83):349-356.

56. Ministry of Health Iraq, Iraq WRsO: Summary: Week 34 ending 24-August 2008. Ministry of Health Iraq. 2008, **Sitrep no. 74**: <https://reliefweb.int/sites/reliefweb.int/files/resources/329E5325F2C5F87C852574C10063268A-Full_Report.pdf>. Accessed November 16, 2020.

57. ProMED-mail.: Cholera, diarrhea & dysentery update 2008 (34). 2008, **Archive Number: 20080904.2765**: <https://promedmail.org/promed-post/?id=108549>. Accessed November 16, 2020.

58. Cholera Task Force-Iraq: Update on Current Cholera Outbreak in Iraq. Cholera Task Force-Iraq. 2015, **Sitrep No 1, Epi Week 37**: <https://reliefweb.int/sites/reliefweb.int/files/resources/SITREP__1__13_Sept_15EXT.pdf>. Accessed November 16, 2020.

59. Mahamud AS, Ahmed JA, Nyoka R, Auko E, Kahi V, Ndirangu J, Nguhi M, Burton JW, Muhindo BZ, Breiman RF *et al*: **Epidemic cholera in Kakuma Refugee Camp, Kenya, 2009: the importance of sanitation and soap**. *J Infect Dev Ctries* 2012, **6**(3):234-241.

60. Abdullahi KN, Mutindin D, Kabugi W, Mowlid S: **Epidemiological Description of a Protracted Cholera Outbreak in Hagadera Refugee Camp and the Surrounding Host Community within Fafi Sub County and Garissa County in Kenya during March-September 2019**. *Epidemiology – Open Journal* 2019, **4**(1):31-35.

61. Kpayli M: Southeasterners suffer another cholera outbreak. Liberian Times. 2008: <https://reliefweb.int/report/liberia/liberia-southeasterners-suffer-another-cholera-outbreak>. Accessed November 16, 2020.

62. UN Mission in Liberia: UNMIL Humanitarian Situation Report. UN Mission in Liberia. 2007, **No. 131**: <https://reliefweb.int/report/liberia/liberia-unmil-humanitarian-situation-report-no-131>. Accessed November 16, 2020.

63. UNICEF: Zoom on Mano River Basin. UNICEF West and Central Africa Regional Office. 2017, **Week 16**: <https://reliefweb.int/sites/reliefweb.int/files/resources/WCA%20Cholera_Update_W16.pdf>. Accessed November 16, 2020.

64. Government of Liberia, WHO: Severe Acute Watery Diarrhea (Cholera). WHO Liberia. 2017, **Epi Week 13**: <https://reliefweb.int/sites/reliefweb.int/files/resources/liberia-early-warning-disease-surveillance-bulletin-week-13.doc.pdf>. Accessed November 16, 2020.

65. ICRC: Mali: preventing the spread of cholera near Gao. International Committee of the Red Cross. 2012: <https://www.icrc.org/en/doc/resources/documents/news-release/2012/mali-news-2012-07-05.htm>. Accessed November 16, 2020.

66. Chen WH, Azman AS: **Mitigating Cholera in the Aftermath of Cyclone Idai**. *Am J Trop Med Hyg* 2019.

67. Kahn R, Mahmud AS, Schroeder A, Aguilar Ramirez LH, Crowley J, Chan J, Buckee CO: **Rapid Forecasting of Cholera Risk in Mozambique: Translational Challenges and Opportunities**. *Prehosp Disaster Med* 2019, **34**(5):557-562.

68. WHO AFRO: Situation Report 1. WHO Regional Office for Africa. 2019, **Sitrep 1**: <https://www.afro.who.int/sites/default/files/2019-05/WHOSitRep1Mozambique06-07-2019.pdf>. Accessed November 16, 2020.

69. Cambaza E, Mongo E, Anapakala E, Nhambire R, Singo J, Machava E: **Outbreak of Cholera Due to Cyclone Kenneth in Northern Mozambique, 2019**. *Int J Environ Res Public Health* 2019, **16**(16).

70. Yadav DK, Tamrakar D, Baral R, Jha P, Gautam S, Pokharel PK: **Outbreak of cholera in Tilathi VDC Saptari Nepal**. *Kathmandu Univ Med J (KUMJ)* 2012, **10**(40):36-39.

71. Roskosky M, Acharya B, Shakya G, Karki K, Sekine K, Bajracharya D, von Seidlein L, Devaux I, Lopez AL, Deen J *et al*: **Feasibility of a Comprehensive Targeted Cholera Intervention in The Kathmandu Valley, Nepal**. *Am J Trop Med Hyg* 2019.

72. Sekine K, Roskosky M: **Lessons Learned from Enhancing Sentinel Surveillance for Cholera in Post-Earthquake Nepal in 2016**. *Am J Trop Med Hyg* 2019, **100**(3):494-496.

73. Yadav S: **Cholera Outbreak in Gaidataar: A Lesson for Further Strengthening the Task Force for Epidemic Management in Nepal**. *JNMA J Nepal Med Assoc* 2017, **56**(207):374-376.

74. UNICEF: Alert: Niger health district Dosso. UNICEF West and Central Africa Regional Office. 2016, **Week 40**: <https://reliefweb.int/sites/reliefweb.int/files/resources/WCA%20Cholera_Update_W40.pdf>. Accessed November 16, 2020.

75. UNICEF: Niger - Dosso. UNICEF West and Central Africa Regional Office. 2016, **Week 42**: <https://reliefweb.int/sites/reliefweb.int/files/resources/WCA%20Cholera_Update_W42.pdf>. Accessed November 16, 2020.

76. WHO AFRO: Cholera outbreak in Niger. WHO Regional Office for Africa. 2018, **Week 29: 14 July-20 July 2018**: <https://apps.who.int/iris/bitstream/handle/10665/273214/OEW29-1420072018.pdf>. Accessed November 16, 2020.

77. Dan-Nwafor CC, Ogbonna U, Onyiah P, Gidado S, Adebobola B, Nguku P, Nsubuga P: **A cholera outbreak in a rural north central Nigerian community: an unmatched case-control study**. *BMC Public Health* 2019, **19**(1):112.

78. Ngwa MC, Wondimagegnehu A, Okudo I, Owili C, Ugochukwu U, Clement P, Devaux I, Pezzoli L, Ihekweazu C, Jimme MA *et al*: **The multi-sectorial emergency response to a cholera outbreak in Internally Displaced Persons camps in Borno State, Nigeria, 2017**. *BMJ Glob Health* 2020, **5**(1):e002000.

79. Hassan A, Mohammed K, Alaka A, Balami K, Njidda A, Malgwi A, Eteng W, Lawal B, Dalhat M, Balogun M *et al*: **Outbreak Investigation of Cholera in an Internally Displaced Persons camp in. Borno State, Northeastern Nigeria –. August 2017**. In: *International Conference on (re-)emerging infectious diseases (ICREID)* Addis Ababa, Ethiopia; 2017.

80. WHO AFRO: Cholera in Borno State, Nigeria. WHO Regional Office for Africa. 2017, **Week 36: 2-8 September 2017**: <https://apps.who.int/iris/bitstream/handle/10665/258922/OEW36-2892017.pdf?sequence=1>. Accessed

81. WHO AFRO: Cholera in north-east Nigeria. WHO Regional Office for Africa. 2018, **Week 11: 10-16 March 2018**: <https://apps.who.int/iris/bitstream/handle/10665/260520/OEW11-101632018.pdf?sequence=1>. Accessed November 16, 2020.

82. **Cholera in Pakistan** [<https://www.who.int/csr/don/2010_10_25/en/>]

83. Disasters Emergency Committee: Cholera Hits Pakistan Three Months After Floods Starts. Disasters Emergency Committee. 2010: <https://www.dec.org.uk/press-release/cholera-hits-pakistan-three-months-after-floods-start>. Accessed November 16, 2020.

84. WHO EMRO: Cholera in Pakistan. WHO Regional Office for the Eastern Mediterranean. 2010, **3**(43): <https://applications.emro.who.int/dsaf/epi/2010/Epi_Monitor_2010_3_43.pdf?ua=1&ua=1>. Accessed November 16, 2020.

85. Akram K: **Investigation of Cholera Outbreak at Rawalpindi, Pakistan - August 2017**. *Iproceedings* 2018, **4**(1):e10579.

86. Rosewell A, Dagina R, Murhekar M, Ropa B, Posanai E, Dutta SR, Jennison A, Smith H, Mola G, Zwi A *et al*: **Vibrio cholerae O1 in 2 coastal villages, Papua New Guinea**. *Emerg Infect Dis* 2011, **17**(1):154-156.

87. International Federation of the Red Cross and Red Crescent: DREF Operation, Papua New Guinea: Cholera, dysentery and influenza outbreaks, DREF operation n° MDRPG004. International Federation of the Red Cross and Red Crescent. 2009(Glide No. EP-2009-000185-PNG): <https://reliefweb.int/sites/reliefweb.int/files/resources/EFAA8C5B1D718088C125762B00415B26-Full_Report.pdf>. Accessed November 16, 2020.

88. **Outbreak news. Cholera, Sierra Leone**

89. WHO EMRO: Cholera in Somalia. WHO Regional Office for the Eastern Mediterranean. 2008, **1**(10): <http://applications.emro.who.int/dsaf/epi/2008/Epi_Monitor_2008_1_10.pdf?ua=1>. Accessed November 16, 2020.

90. UNICEF, OCHA: Brief preliminary report of UNICEF and OCHA AWD/suspected cholera fact finding mission to Balet Xaawo (as of 12 June 2015). UNICEF. 2015: <https://reliefweb.int/sites/reliefweb.int/files/resources/160615_brief_report_of_the_balet_xaawo_unicef_and_ocha_mission.pdf>. Accessed November 16, 2020.

91. Ministry of Health Somalia, WHO: Situation Report for Acute Watery Diarrhoea/Cholera. Ministry of Health Somalia. 2017, **Epidemiological Week 50 (11th to 17th Dec 2017)**: <https://reliefweb.int/sites/reliefweb.int/files/resources/week_50_awd_cholera_sitrep_final_0.pdf>. Accessed November 16, 2020.

92. WHO EMRO: New Cholera Outbreak in Somalia. WHO Regional Office for the Eastern Mediterranean. 2018, **11**(6): <http://applications.emro.who.int/docs/epi/2018/Epi_Monitor_2018_11_06.pdf?ua=1>. Accessed November 16, 2020.

93. International Federation of the Red Cross and Red Crescent: Sudan: Cholera Final report, Emergency appeal n° MDRSD001. International Federation of the Red Cross and Red Crescent. 2008: <https://reliefweb.int/sites/reliefweb.int/files/resources/F1F7E51BB603CAA7852574AC00580E32-Full_Report.pdf>. Accessed November 16, 2020.

94. Dunoyer J, Moore S, Valingot C, Rebaudet S, Gaudart J, Piarroux R, Sudre B: Epidemiological study of cholera hotspots and epidemiological basins in East and Southern Africa: In-depth cholera epidemiological report for South Sudan. Prospective Cooperation and UNICEF. 2018: <https://plateformecholera.info/attachments/article/639/Cholera%20epidemiology%20in%20South%20Sudan_UNICEF_April%202018_FINAL.pdf>. Accessed November 16, 2020.

95. Medair: Sudan: Cholera intervention in Yei. Medair. 2008: <https://reliefweb.int/report/sudan/sudan-cholera-intervention-yei>. Accessed November 16, 2020.

96. WHO EMRO: Cholera outbreak in southern Sudan. 2008, **1**(22 & 23): <http://applications.emro.who.int/dsaf/epi/2008/Epi_Monitor_2008_1_22-23.pdf?ua=1>. Accessed

97. Abubakar A, Bwire G, Azman AS, Bouhenia M, Deng LL, Wamala JF, Rumunu J, Kagirita A, Rauzier J, Grout L *et al*: **Cholera Epidemic in South Sudan and Uganda and Need for International Collaboration in Cholera Control**. *Emerg Infect Dis* 2018, **24**(5):883-887.

98. WHO Emergencies PaR: Cholera outbreak, South Sudan. WHO. 2014: <https://www.who.int/csr/don/2014_05_30/en/>. Accessed November 16, 2020.

99. Republic of South Sudan, Ministry of Health: Cholera Outbreak in Juba, Republic of South Sudan. Ministry of Health of South Suda. 2014: <https://reliefweb.int/sites/reliefweb.int/files/resources/Juba-Cholera-15-May-2014.pdf>. Accessed November 16, 2020.

100. Ministry of Health SS, WHO: Cholera in Juba, Central Equatoria State, Republic of South Sudan. Ministry of Health of South Sudan. 2015, **Situation Report # 1**: <https://www.who.int/hac/crises/ssd/sitreps/south_sudan_cholera_22june2015.pdf?ua=1>. Accessed November 16, 2020.

101. Republic of South Sudan, Ministry of Health: Suspect cholera. Ministry of Health of South Sudan. 2016, **W26, 27 June-3 July, 2016**: <https://www.who.int/hac/crises/ssd/south_sudan_epi_3july2016.pdf>. Accessed November 16, 2020.

102. Nsubuga F, Garang SC, Tut M, Oguttu D, Lubajo R, Lodiongo D, Lasuba M, Mpairwe A: **Epidemiological description of a protracted cholera outbreak in Tonj East and Tonj North counties, former Warrap State, South Sudan, May-Oct 2017**. *BMC Infect Dis* 2019, **19**(1):4.

103. Health Cluster Sudan: Health Cluster Bulletin: the Cholera response overview (September – December 2019). Health Cluster Sudan,. 2019: <https://reliefweb.int/sites/reliefweb.int/files/resources/hc_bulletin_december_2019-_cholera_outbreak_reposne_overview.pdf>. Accessed November 16, 2020.

104. WHO Emergencies PaR: Cholera – Republic of the Sudan. WHO. 2019: <https://www.who.int/csr/don/15-october-2019-cholera-republic-of-the-sudan/en/>. Accessed November 16, 2020.

105. Sudan Humanitarian Country Team: Sudan: Humanitarian cholera readiness and response [Activities to support National AWD Preparedness and Response Plan 2018-19]. Sudan Humanitarian Country Team. 2019: <https://reliefweb.int/sites/reliefweb.int/files/resources/191001_Consolidated%20Readiness%20and%20Response%20Plan%20for%20Cholera.pdf>. Accessed November 16, 2020.

106. WHO EMRO: OCV campaign Sudan. WHO Regional Office for the Eastern Mediterranean. 2019, **12**(44): <https://applications.emro.who.int/docs/epi/2019/22244220-2019-12-44.pdf?ua=1&ua=1>. Accessed November 16, 2020.

107. Assistance Coordination Unit: Report on suspected case of Cholera in N Syria: EWARN Report. Assistance Coordination Unit. 2015: <https://www.acu-sy.org/en/suspected-case-of-cholera-in-n-syria/>. Accessed November 16, 2020.

108. Assistance Coordination Unit: Epidemiological Weekly Bulletin Syria - Early Warning Alert and Response Network

Assistance Coordination Unit. 2015, **Epiweek No. 42**: <https://www.acu-sy.org/en/epi-reports/?b5-file=1954&b5-folder=606>. Accessed November 16, 2020.

109. Assistance Coordination Unit: Epidemiological Weekly Bulletin - Syria Early Warning Alert and Response Network (EWARN). Assistance Coordination Unit. 2017, **Epiweek 42**: <https://www.acu-sy.org/en/epi-reports/?b5-file=4540&b5-folder=606>. Accessed November 16, 2020.

110. ProMED-mail: Cholera, diarrhea & dysentery update (122): Asia (Syria). ProMED-mail. 2017, **Archive Number: 20171021.5394037**: <https://promedmail.org/promed-post/?id=5394037>. Accessed November 16, 2020.

111. Narra R, Maeda JM, Temba H, Mghamba J, Nyanga A, Greiner AL, Bakari M, Beer KD, Chae SR, Curran KG *et al*: **Notes from the Field: Ongoing Cholera Epidemic - Tanzania, 2015-2016**. *MMWR Morb Mortal Wkly Rep* 2017, **66**(6):177-178.

112. WHO: WHO notified of new cholera outbreaks in Tanzania. WHO. 2015: <https://reliefweb.int/report/united-republic-tanzania/who-notified-new-cholera-outbreaks-tanzania>. Accessed November 16, 2020.

113. UNHCR: Burundi situation, UNHCR regional update, No. 0013. UNHCR. 2015, **No. 0013**: <https://reliefweb.int/sites/reliefweb.int/files/resources/UNHCR_BurundiSituation_No0013.pdf>. Accessed November 16, 2020.

114. UNHCR: Burundi situation, UNHCR regional update, No. 0011. UNHCR. 2015, **No. 0011**: <https://data2.unhcr.org/en/documents/download/48620>. Accessed November 16, 2020.

115. Bwire G, Malimbo M, Kagirita A, Makumbi I, Mintz E, Mengel MA, Orach CG: **Nosocomial Cholera Outbreak in a Mental Hospital: Challenges and Lessons Learnt from Butabika National Referral Mental Hospital, Uganda**. *Am J Trop Med Hyg* 2015, **93**(3):534-538.

116. Kwesiga B, Pande G, Ario AR, Tumwesigye NM, Matovu JKB, Zhu BP: **A prolonged, community-wide cholera outbreak associated with drinking water contaminated by sewage in Kasese District, western Uganda**. *BMC Public Health* 2017, **18**(1):30.

117. Pande G, Kwesiga B, Bwire G, Kalyebi P, Riolexus A, Matovu JKB, Makumbi F, Mugerwa S, Musinguzi J, Wanyenze RK *et al*: **Cholera outbreak caused by drinking contaminated water from a lakeshore water-collection site, Kasese District, south-western Uganda, June-July 2015**. *PLoS One* 2018, **13**(6):e0198431.

118. WHO: Cholera in Uganda. WHO Regional Office for Africa. 2018, **Week 8: 17-23 February 2018**: <https://apps.who.int/iris/bitstream/handle/10665/260335/OEW8-1723022018.pdf?sequence=1>. Accessed November 16, 2020.

119. WHO EMRO: Cholera in Yemen. WHO Regional Office for the Eastern Mediterranean. 2010, **3**(27): <http://applications.emro.who.int/dsaf/epi/2010/Epi_Monitor_2010_3_27.pdf?ua=1>. Accessed November 16, 2020.

120. WHO EMRO: Cholera in Yemen. WHO Regional Office for the Eastern Mediterranean. 2011, **4**(42): <http://applications.emro.who.int/dsaf/epi/2011/Epi_Monitor_2011_4_42.pdf?ua=1>. Accessed November 16, 2020.

121. WHO EMRO: Humanitarian Health Action: Growing needs for emergency life-saving health care in Yemen, Monthly Highlights. WHO Regional Office for the Eastern Mediterranean. 2011: <https://www.who.int/hac/crises/yem/highlights/june2011/en/>. Accessed November 16, 2020.

122. Qasem MA, Al-Abhar N, Jumaan A: The Hazard of conflict: cholera outbreak in Abyan, Yemen, 2011. EMPHNET. 2014: <http://www.yfetp.com/OPSHandler/Resources/Attachment/RoxyFileman//First%20National%20YFETP%20Conference%20Book%20Feb%202014.pdf>. Accessed November 16, 2020.

123. **Evaluation of the UNICEF Level 3 Response to the Cholera Epidemic in Yemen: A Crisis within a Crisis**

124. WHO EMRO: The Ministry of Public Health and Population announces cholera cases in Yemen. WHO Regional Office for the Eastern Mediterranean. 2016: <http://www.emro.who.int/media/news/the-ministry-of-health-announces-cholera-cases-in-yemen.html>. Accessed November 16, 2020.

125. Sinyange N, Brunkard JM, Kapata N, Mazaba ML, Musonda KG, Hamoonga R, Kapina M, Kapaya F, Mutale L, Kateule E *et al*: **Cholera Epidemic - Lusaka, Zambia, October 2017-May 2018**. *MMWR Morb Mortal Wkly Rep* 2018, **67**(19):556-559.

126. WHO: Cholera – Zambia. WHO. 2017: <https://www.who.int/csr/don/11-december-2017-cholera-zambia/en/>. Accessed November 16, 2020.

127. Ministry of Health Z: Cholera Update. Ministry of Health, Zambia. 2017: <https://www.moh.gov.zm/?p=5394>. Accessed November 16, 2020.

128. WHO AFRO: Cholera in Zambia. WHO Regional Office for Africa. 2017, **Week 41: 7-13 October 2017**: <https://apps.who.int/iris/bitstream/handle/10665/259263/OEW41-713102017.pdf?sequence=1>. Accessed November 16, 2020.

129. **Situational Report No. 13** [<https://reliefweb.int/sites/reliefweb.int/files/resources/Cholera%20Sitrep%20as%20at%20%2002_04_19%20%284%29%20sit%20rep%2013_0_0.pdf>]

130. WHO: Cholera in Zimbabwe: Epidemiological Bulletin number 1. WHO Zimbabwe. 2008: <https://reliefweb.int/sites/reliefweb.int/files/resources/392C76417DE84B01C125752100327DBE-Full_Report.pdf>. Accessed November 16, 2020.

131. WHO: Cholera in Zimbabwe: Epidemiological Bulletin number 2. WHO Zimbabwe. 2008: <https://reliefweb.int/sites/reliefweb.int/files/resources/E939C04030DA4F194925752C0017AB9B-Full_Report.pdf>. Accessed November 16, 2020.

132. Médecins Sans Frontières: Sewage problems cause cholera outbreak in Zimbabwe. Médecins Sans Frontières. 2008: <https://www.msf.org/sewage-problems-cause-cholera-outbreak-zimbabwe>. Accessed November 16, 2020.

133. UNICEF: Zimbabwe: Cholera outbreak report. UNICEF Zimbabwe. 2018, **Sitrep 1**: <https://reliefweb.int/sites/reliefweb.int/files/resources/UNICEF%20Zimbabwe%20Cholera%20Situation%20Report%20-13%20September%202018.pdf>. Accessed November 16, 2020.

134. WHO Emergencies PaR: Cholera – Zimbabwe. WHO. 2018: <https://www.who.int/csr/don/20-september-2018-cholera-zimbabwe/en/>. Accessed November 16, 2020.

135. WHO AFRO: Cholera in Zimbabwe. WHO Regional Office for Africa. 2018, **Week 36: 1-7 September 2018**: <https://apps.who.int/iris/bitstream/handle/10665/274335/OEW36-15092018.pdf>. Accessed November 16, 2020.

136. McAteer JB, Danda S, Nhende T, Manamike P, Parayiwa T, Tarupihwa A, Tapfumanei O, Manangazira P, Mhlanga G, Garone DB *et al*: **Notes from the Field: Outbreak of Vibrio cholerae Associated with Attending a Funeral - Chegutu District, Zimbabwe, 2018**. *MMWR Morb Mortal Wkly Rep* 2018, **67**(19):560-561.

137. Chimusoro A, Maphosa S, Manangazira P, Phiri I, Nhende T, Danda S, Tapfumanei O, Munyaradzi Midzi S, Nabyonga-Orem J: **Responding to Cholera Outbreaks in Zimbabwe: Building Resilience over Time**. In: *Current Issues in Global Health.* edn. Edited by David Claborn; 2018.

138. WHO AFRO: Cholera in Zimbabwe. WHO Regional Office for Africa. 2018, **Week 4: 20-26 January 2018**: <http://apps.who.int/iris/bitstream/10665/259942/1/OEW4-202612018.pdf>. Accessed November 16, 2020.
