# Supplementary figures and images for: Early detection of cholera epidemics to support control in fragile states: estimation of delays and potential epidemic sizes
Source: BMC Med. 2020 Dec 15;18:397. doi: 10.1186/s12916-020-01865-7 (PMC7737284; doi:10.1186/s12916-020-01865-7)

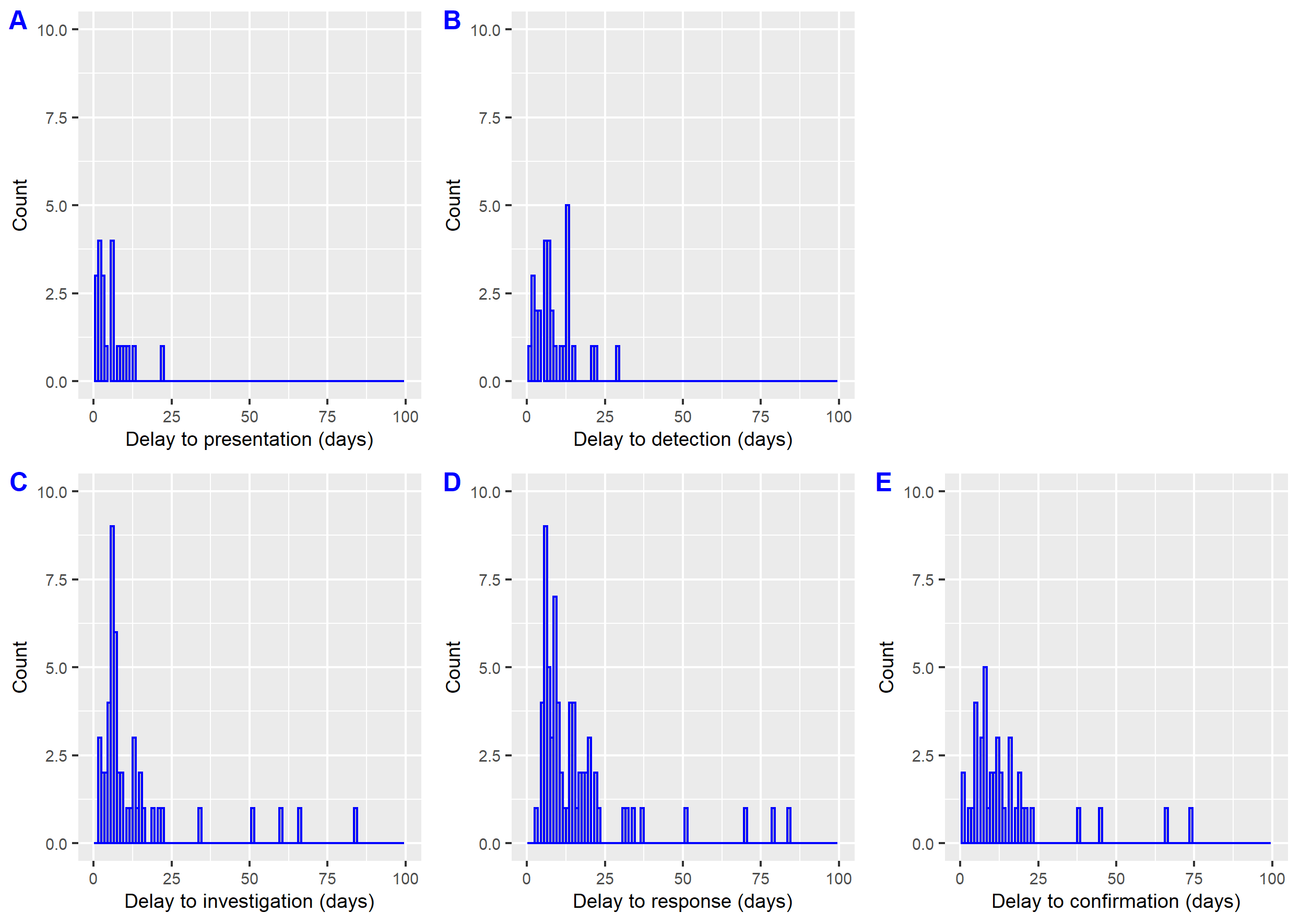

Supplement: Supplementary file 3 — Additional file 3. Histograms of delays from symptom onset to (A) case presentation, (B) outbreak detection, (C) investigation, (D) response, and (E) confirmation. [file 12916_2020_1865_MOESM3_ESM.tiff]
