# Supplementary material for: Early detection of cholera epidemics to support control in fragile states: estimation of delays and potential epidemic sizes
Source: BMC Med. 2020 Dec 15;18:397. doi: 10.1186/s12916-020-01865-7 (PMC7737284; doi:10.1186/s12916-020-01865-7)
Supplement: Supplementary file 4 — Additional file 4. Overview of alternative models for the main analyses. [file 12916_2020_1865_MOESM4_ESM.docx]

**Additional file 4**

**Overview of alternative models for the main analyses**

**Main analyses: model selection using Akaike Information Criterion (AIC)**

| Model | Signal | Year | Region | Context | Crisis | Parameters | AIC |
| --- | --- | --- | --- | --- | --- | --- | --- |
| m1 | 0 | 1 | 1 | 1 | 1 | 4 | -33.2 |
| m2 | 0 | 1 | 0 | 1 | 1 | 3 | -39.0 |
| m3 | 0 | 1 | 0 | 1 | 0 | 2 | -41.7 |
| *m4** | 0 | 1 | 0 | 0 | 0 | 1 | -45.6 |

*Model used in the main analysis.

**Model parameters (Y= delay from onset of symptoms to response)**

| Model | N_Y_ | X | N_X_ | Est | % change | %  LCI | % UCI | SE | *p*,  Est | Adj. r^2^ | F-stat | *p*,  F-stat |
| --- | --- | --- | --- | --- | --- | --- | --- | --- | --- | --- | --- | --- |
| m4 | 67 | Year | 76 | -0.05 | -5.18 | -9.61 | -0.52 | 0.02 | 0.03 | 0.06 | 4.90 | 0.03 |
| m5 | 67 | Alert | 49 | -0.50 | -40.24 | -60.99 | -5.67 | 0.22 | 0.03 | 0.08 | 5.19 | 0.03 |

LCI, lower 95% confidence interval, UCI, upper 95% confidence interval, SE, standard error
