# Supplementary material for: Early detection of cholera epidemics to support control in fragile states: estimation of delays and potential epidemic sizes
Source: BMC Med. 2020 Dec 15;18:397. doi: 10.1186/s12916-020-01865-7 (PMC7737284; doi:10.1186/s12916-020-01865-7)
Supplement: Supplementary file 5 — Additional file 5. Model parameters for additional delay analyses. [file 12916_2020_1865_MOESM5_ESM.docx]

**Additional file 5**

**Model parameters for additional delay analyses (X=year)**

| Model (Y) | N_Y_ | N_X_ | Est | % change | %  LCI | %  UCI | SE | *p*,  Est | Adj. r^2^ | F-stat | *p*,  F-stat |
| --- | --- | --- | --- | --- | --- | --- | --- | --- | --- | --- | --- |
| Presentation | 76 | 76 | -0.02 | -1.87 | -5.40 | 1.80 | 0.02 | 0.31 | 0.00 | 1.05 | 0.31 |
| Detection | 76 | 76 | -0.05 | -4.66 | -8.46 | -0.69 | 0.02 | 0.02 | 0.06 | 5.43 | 0.02 |
| Investigation | 48 | 76 | -0.10 | -9.42 | -15.88 | -2.47 | 0.04 | 0.01 | 0.12 | 7.25 | 0.01 |
| Response | 67 | 76 | -0.05 | -5.18 | -9.61 | -0.52 | 0.02 | 0.03 | 0.06 | 4.90 | 0.03 |
| Confirmation | 41 | 76 | -0.10 | -9.57 | -16.10 | -2.53 | 0.04 | 0.01 | 0.14 | 7.37 | 0.01 |

LCI, lower 95% confidence interval, UCI, upper 95% confidence interval, SE, standard error
